# Thermogenesis in Adipose Tissue: Adrenergic and Non-Adrenergic Pathways

**DOI:** 10.3390/cells15020131

**Published:** 2026-01-12

**Authors:** Md Arafat Hossain, Ankita Poojari, Atefeh Rabiee

**Affiliations:** Department of Pharmaceutical Sciences, Thomas J. Long School of Pharmacy, University of the Pacific, Stockton, CA 95211, USA; m_hossain@u.pacific.edu (M.A.H.); a_poojari@u.pacific.edu (A.P.)

**Keywords:** non-shivering thermogenesis, brown adipose tissue, beige adipocytes, UCP1-independent mechanisms, β2 vs. β3 adrenoceptors, energy expenditure, obesity therapy, futile cycles

## Abstract

Obesity has reached epidemic proportions, driven by energy imbalance and limited capacity for adaptive thermogenesis. Brown (BAT) and beige adipose tissues dissipate energy through non-shivering thermogenesis (NST), primarily via uncoupling protein-1 (UCP1), making them attractive targets for increasing energy expenditure (EE). The canonical β-adrenergic pathway robustly activates NST in rodents through β3 adrenoceptors; however, translational success in humans has been limited by low β3 expression, off-target cardiovascular effects, and the emerging dominance of β2-mediated signaling in human BAT. Consequently, attention has shifted to non-adrenergic and UCP1-independent mechanisms that offer greater tissue distribution and improved safety profiles. This review examines a broad spectrum of alternative receptors and pathways—including GPRs, TRP channels, TGR5, GLP-1R, thyroid hormone receptors, estrogen receptors, growth hormone, BMPs, sirtuins, PPARs, and interleukin signaling—as well as futile substrate cycles (Ca^2+^, creatine, and glycerol-3-phosphate) that sustain thermogenesis in beige adipocytes and skeletal muscle. Pharmacological agents (natural compounds, peptides, and small molecules) and non-pharmacological interventions (cold exposure, exercise, diet, and time shift) targeting these pathways are critically evaluated. We highlight the translational gaps between rodent and human studies, the promise of multimodal therapies combining low-dose adrenergic agents with non-adrenergic activators, and emerging strategies such as sarco/endoplasmic reticulum calcium ATPase protein (SERCA) modulators and tissue-specific delivery. Ultimately, integrating adrenergic and non-adrenergic approaches holds the greatest potential for safe, effective, and sustainable obesity management.

## 1. Introduction

Obesity has been rising uncontrollably for decades, turning it into a global epidemic. The WHO reports that adult obesity has tripled and adolescent obesity quadrupled since 1975; 41.9% of US adults are obese [1]. In the US, obesity-related costs exceed USD 425.5 billion annually (2023 estimate) [2]. Obesity, defined as a Body Mass Index (BMI) of 30 kg/m^2^ or more, constitutes a complex, multifactorial disease rather than a simple condition. It is affected by numerous factors, including environmental, social, behavioral, cultural, psychological, metabolic, and genetic influences. Beyond esthetic considerations, these alterations have a profound impact on mental health and lifespan. Obesity increases the risk of comorbidities such as type 2 diabetes mellitus (T2DM), hypertension, cardiovascular disease (CVDs), certain cancers, and osteoarthritis [3,4]. Although obesity affects all groups of people, it is more prevalent in women and adults.

Obesity results from an imbalance between energy intake and expenditure. When energy intake exceeds expenditure, excess energy is stored as triglycerides (TGs) in white adipose tissue (WAT). Mammals have two classical types of fat cells: white and brown. Unlike white fat, brown fat is designed to burn energy and produce heat (thermogenesis). It does this by breaking down TGs via β-oxidation. The key protein responsible is UCP1, located in the inner mitochondrial membrane, which “short-circuits” ATP to release heat. While brown adipose tissue (BAT) has high mitochondrial content and UCP1 expression, WAT differs, having fewer mitochondria and lower UCP1 expression. However, the presence of inducible thermogenic adipocytes within WAT has been discovered, known as beige or “brite” fat, which exhibits intermediate mitochondrial density and UCP1 expression. Recent research has revealed the presence of a substantial amount of beige fat in healthy and lean individuals. Inducing browning in white fat decreases the adverse effects of excess WAT and improves overall metabolic health [5]. For this reason, beige fat is an emerging target of interest for many researchers seeking therapeutic interventions for obesity and other metabolic diseases.

The synthesis and oxidation of fatty acids (FAs) are stimulated and tightly regulated by adrenergic activation. Upon adrenergic stimulation, brown or beige adipocyte lipolysis and mitochondrial respiration are activated in a UCP1-dependent and/or independent manner. The β-adrenergic pathway, activated by cold exposure and exercise, primarily regulates NST through UCP1. In rodents, β3AR is predominantly expressed in BAT and drives thermogenesis via cAMP-PKA, whereas in humans, BAT expression is low [6,7]. Initial studies performed in rodents demonstrated the importance of the β3 receptor in thermogenesis; however, surprisingly, these results did not translate well to humans. Consequently, β3-receptor agonists have historically performed poorly in human clinical trials [6,8,9]. Mirabegron, a selective β3 agonist effective in obese rodents, failed to reproduce comparable metabolic benefits in humans despite initial promise [6,8,9]. Although it increased thermogenesis, this effect occurred only at high doses and with chronic use. It was associated with unintended cardiovascular side effects, as high doses of mirabegron activated other β subtypes [8]. This lower efficacy in humans was attributed to lower β3-receptor expression in humans as compared to rodents [7]. In addition, much of what is known about human BAT function comes from studies of cold-stimulated conditions, in which thermogenesis and substrate clearance are mainly driven by shivering skeletal muscles. Although controversial, recent research suggests that the β2 receptor plays a more important role in thermogenesis and EE in humans [8]. However, what truly hinders its potential as an attractive anti-obesity target is its widespread presence across multiple organs, which causes unwanted side effects alongside thermogenesis. β2-selective agonists (e.g., formoterol, salbutamol) increase EE but lack BAT specificity. The adrenergic pathways that regulate thermogenesis remain widely studied, but recent research has now shifted toward noncanonical pathways as safer and more effective options for obesity treatment. This review explores innovative non-adrenergic strategies to address these challenges, paving the way for safer, multimodal obesity therapies. In this review, a defined translational framework is applied to evaluate evidence of thermogenesis across species. Changes in body weight, lipid content, EE, heat generation, glucose uptake, and insulin sensitivity are considered primary endpoints. Changes in different thermogenic markers, such as UCP1, DIO2, PPARγ, PRDM16, and PGC1α, are considered secondary endpoints. Mechanisms supported only by rodent studies are interpreted as hypothesis-generating rather than therapy-ready without validation in human tissues or clinical studies.

## 2. Adipose Tissue Dysfunction and Types of Fat

Adipose tissue (AT) is now recognized as an important endocrine organ. Beyond fat storage and EE, AT regulates whole-body energy homeostasis, insulin sensitivity, and immune function. For many years, it was viewed as a simple, inert storage site for excess energy. Over the past two decades, research has revealed remarkable heterogeneity and plasticity in this system, particularly in response to physiological stimuli such as cold exposure and intermittent fasting. AT consists of adipocytes and a stromal vascular fraction (SVF) containing endothelial cells, immune cells, and mesenchymal stem cells (MSCs). These components interact in a feedback loop to maintain metabolic health. In pathological conditions such as obesity, adipocytes undergo hypertrophy (enlargement of existing adipocytes) and hyperplasia (generation of new, mature adipocytes to capture circulating excess lipids), thereby altering their secretory profile [10]. Hormones regulate the hyperplastic expansion of AT in satiety, hunger, metabolism, and activity through endocrine, paracrine, autocrine, and neural mechanisms. Key central players include GLP-1, neuropeptide Y, leptin, ghrelin, and cholecystokinin (CCK), while insulin, PPARγ ligands, retinoids, corticosteroids, and tumor necrosis factor-alpha (TNF-α) work as peripheral regulators [11]. AT is innervated by sympathetic neurons, with adenomatous polyposis coli (APC) proliferation responsive to β-adrenergic signaling [11]. Larger adipocytes are associated with worse tissue function, increased local inflammation, and reduced lipid storage [11]. The immune cell composition within AT is geared towards an inflammatory phenotype, with remodeling of the extracellular matrix exacerbating metabolic dysfunction [12]. Beyond metabolic disorders, AT dysfunction is also a prominent feature of cancer, where tumor-derived signals profoundly alter adipose metabolism and thermogenic programs (discussed in the section Thermogenesis and AT Browning in Cancer-Associated Adipose Dysfunction) [13].

In mammals, AT forms two types of fat depots, white and brown, distinguished by their location and specialized function. WAT’s function is to store excess metabolic energy as TGs in lipid droplets and to provide thermal insulation and cushioning to most of the subcutaneous and visceral regions. WAT has large lipid droplets and few mitochondria, which are the primary factors explaining its white color. In contrast, BAT appears brownish in hue due to the iron cofactors of the oxidative phosphorylation (OXPHOS) complex, which can be attributed to the higher number of mitochondria and several smaller lipid droplets interspersed throughout the cytoplasm [14]. BAT specializes in energy dissipation and thermogenesis and differentiates before birth, enabling newborns to generate heat and protect themselves from cold exposure. In humans, BAT is found in the supraclavicular, axillary, neck, periaortic, paravertebral, perirenal, and mediastinal regions. BAT volume and activity decline with aging and obesity, making classical BAT a challenging and somewhat controversial therapeutic target for adults. White adipocytes originate from stromal vascular fraction cells that express Sca1, CD34, and CD29, but lack CD31 and CD45 [15]. Conversely, brown adipocytes are developmentally related to skeletal muscle, arising from progenitor cells that express myogenic factor 5 (Myf5) and paired box 7 (Pax7) proteins [16,17].

Besides classical white and brown fat, a third type—beige/bright/brown-in-white fat—can emerge within white adipose depots. Inducible beige adipocytes are particularly abundant in gluteal and femoral subcutaneous fat in humans. Their appearance, known as “browning,” is triggered by external stimuli such as cold exposure, exercise, certain diets, or β-adrenergic drugs. The transformation from white to beige can occur via two different pathways: de novo differentiation from preadipocytes and trans-differentiation of existing mature adipocytes [18,19]. However, it is worth noting that the white-to-beige (browning) transition is reversible upon stimulus withdrawal. Beige adipocyte markers include CD137, PAT2, CITED1, TMEM26, and TBX1 [20,21]. From an evolutionary perspective, beige adipocytes are most similar to white adipocytes, as both develop from mesodermal stem cells that lack the myogenic transcription factor 5 (Myf5) [22]. However, functionally, beige adipocytes closely resemble brown adipocytes, with similar mitochondrial content and the capacity to convert chemical energy into heat in response to specific stimuli [22]. Thus, beige fat represents the primary inducible source of NST in adult humans, where classical BAT is scarce. This explains the growing research focus on safe pharmacological strategies to promote browning of WAT.

### Thermogenesis and AT Browning in Cancer-Associated Adipose Dysfunction

AT thermogenesis and browning have been increasingly implicated in cancer-associated adipose dysfunction. Excess adiposity promotes tumor growth through AT remodeling, with the secretion of certain adipokines and cytokines, such as leptin, TNF-α, IL-6, and monocyte chemoattractant protein-1 (MCP-1), acting as protumor factors [13]. Tumor cells exploit this altered microenvironment through enhanced lipid transfer, glycolytic reprogramming, and stromal interactions, aiding in cancer development and metastasis [13]. This relationship forms a feedback loop: while tumors induce AT remodeling to extract nutrients and signaling molecules, the resulting metabolic imbalances (such as IR and systemic inflammation) further drive tumor aggressiveness.

Cancer-associated cachexia is the most well-characterized link between thermogenesis and cancer. Cachexia is a waste syndrome marked by systemic inflammation, elevated EE, and progressive loss of AT and lean mass. Preclinical studies demonstrate that tumor-derived factors such as pro-inflammatory cytokines, parathyroid-hormone-related protein (PTHrP), and enhanced sympathetic outflow can induce browning-like transcriptional programs in WAT, characterized by increased mitochondrial biogenesis and UCP1 expression [23,24]. This activation is largely mediated through adrenergic signaling pathways and is often accompanied by accelerated lipolysis and hypermetabolism [23,24]. Unlike adaptive browning during cold exposure or obesity, this thermogenic activation occurs in a catabolic inflammatory milieu and contributes to pathological energy wasting rather than metabolic benefit.

These findings highlight the context-dependent nature of thermogenic pathways. Adipose thermogenesis may be beneficial in obesity and metabolic disease, but in cancer, its activation could worsen energy imbalance, emphasizing the need for caution in applying thermogenic strategies across diseases.

## 3. Mitochondria and Thermogenesis

Mitochondria are powerhouse organelles playing a key role in cellular respiration and energy generation. Mitochondria convert nutrients (glucose and FAs) into ATP via the Krebs cycle, electron transport chain, and OXPHOS. With appropriate stimulus, for example, adrenergic activation, long-chain FAs are released, which alter the permeability of the inner mitochondrial membrane to protons. This allows protons to leak into the mitochondrial matrix independent of ATP synthase and causes energy release in the form of heat [25]. In contrast, purine nucleotides (such as ATP, ADP, and GTP) induce a conformational state of UCP1 that is impermeable to protons, serving as a braking system to prevent excessive EE [26]. Although beige adipocytes rely on UCP1 for thermogenesis like BAT, a significant portion of thermogenesis in this fat type is driven by creatine, glycerolipids, and calcium cycling. While calcium and glycerolipid cycling mainly involve participation by the endoplasmic reticulum (ER), creatine cycling occurs in the mitochondria. An enzyme, creatine kinase, phosphorylates creatine to phosphocreatine by hydrolyzing ATP. The resulting phosphocreatine is hydrolyzed by tissue-nonspecific alkaline phosphatase (TNAP) to creatine using ATP, thus creating a futile cycle that consumes ATP and releases energy as heat [27]. Mitochondria in brown and beige adipocytes not only drive thermogenesis but also secrete bioactive molecules. These include neuregulin 4 (NRG4; promotes sympathetic innervation), the lipokine 12,13-diHOME (acts as fuel and signaling molecule), cardiolipin, cytokines, and hormones that further enhance thermogenic capacity. These secreted factors indirectly drive adipocyte maturation, respiration, and angiogenesis, highlighting the key role of mitochondria in thermogenesis.

Apart from these well-studied phenomena, newer mechanisms in which mitochondria play a key role in thermogenesis remain an area of intense research. It has been shown that WAT in subcutaneous depots transfers mitochondria to macrophages, and that these macrophages, when depleted, impair thermogenesis [28]. Another study showed that soluble ADCY10 (adenylate cyclase 10) becomes activated by increased isocitrate dehydrogenase activity in BAT. It facilitates thermogenesis by sustaining mitochondrial membrane potential and complex I activity via cAMP-EPAC1 signaling [29]. Ferroptosis is a lipophilic form of programmed cell death. Mitochondria are especially vulnerable to reactive oxygen species (ROS) and iron metabolism and thus are key sites for ferroptosis. During thermogenesis, iron metabolism is upregulated in BAT, which should make it especially vulnerable to lipid-peroxidation-induced cell death/ferroptosis. BAT may resist ferroptosis via Nfe211 (Nuclear factor erythroid-2-like-1)-mediated proteasomal activation (preliminary evidence) [30]. These recent findings indicate the multifaceted role of mitochondria in promoting or maintaining thermogenesis.

Brown and beige adipocytes contain mitochondria that are small and fragmented in appearance, whereas white adipocytes possess elongated mitochondria that are geared more towards ATP synthesis [31]. Mitochondrial dynamics, in terms of number, size, and motility, have been found to directly affect thermogenesis. Mitochondrial shape is determined by the interplay between fission and fusion events. Fission leads to the formation of small, separate mitochondria, while fusion creates large, interconnected mitochondrial networks. Interplay of outer mitochondrial proteins (Mitofusin 1 (Mfn1) and Mitofusin 2 (Mfn2)) and an inner mitochondrial protein (Optic Atrophy-1 (OPA1)) drives mitochondrial fusion [32]. The key player in mitochondrial fission is Drp1 (dynamin-related protein 1), which is recruited to the outer mitochondrial membrane (OMM) by adaptors/receptors present on the OMM, thereby ensuring fission and increasing mitochondrial content in brown and beige adipocytes [33,34].

## 4. Pathways to Induce Thermogenesis

**Figure 1:**
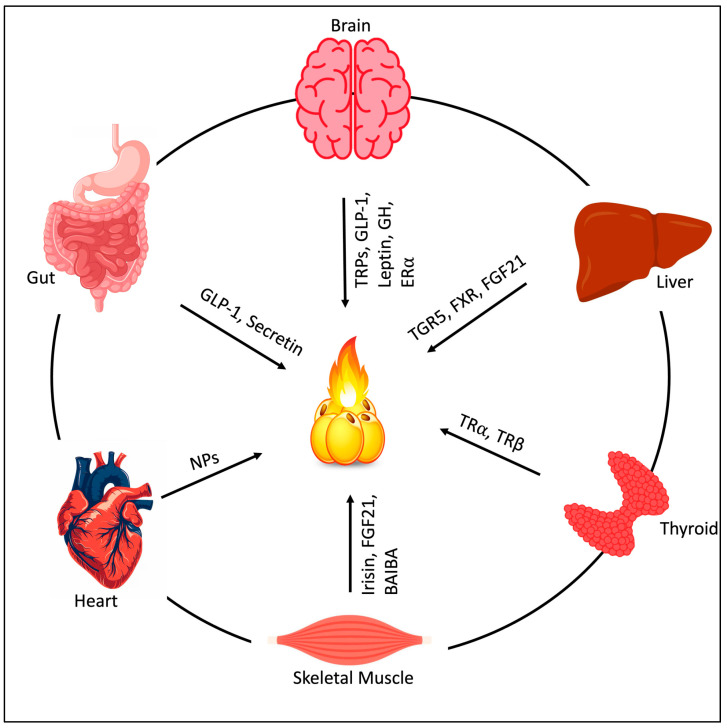
Interactions between thermogenic AT and various organs. Multiple organs, including the brain, liver, muscle, heart, thyroid, and gastrointestinal tract (GIT), crosstalk with thermogenic fat depots. These organs contribute to thermogenesis through receptor activation and the secretion of diverse molecules, such as peptide hormones, lipokines, glucocorticoids, and bile acids. In the figure, the black arrow indicates direct communication between thermogenic fat and different organs to induce thermogenesis.

**Figure 2:**
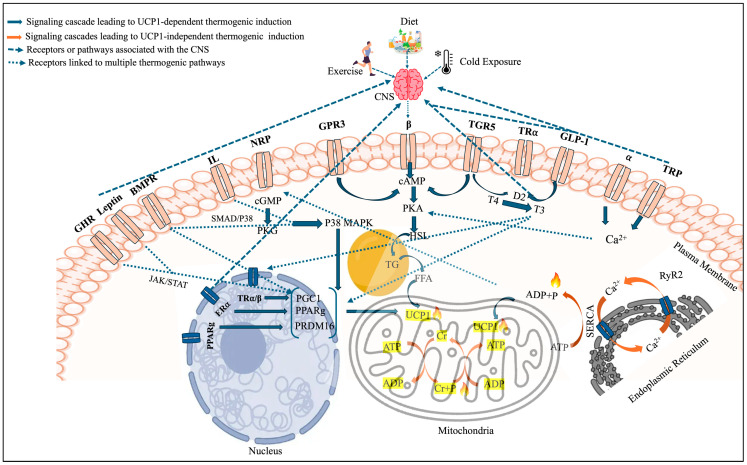
Mechanism of receptor-dependent thermogenic activation. Activation of transmembrane receptors such as β-AR, α-AR, TRPs (TRPV1, TRPV2, TRPV4, TRPM2, TRPM8, TRPA1), TGR5, GLP-1, GHR, NPR, and GPR3, and nuclear receptors such as TRa and Era, activates cellular signaling cascades (PKA, PKG, Sirt1, SMAD, JAK/STAT, AMPK, and p38 MAPK), transcriptional regulators (PRDM family, PGC1α, PPAR family and, Zfp516), and cytokines (IL-4 and IL-13) to increase UCP1 expression in mitochondria to induce thermogenesis in a UCP1-dependent manner. TRa stimulates the conversion of T4 to T3 by stimulating the activity of DIO2, and T3 boosts thermogenesis both via local (TRa/β) and central (via β3) mechanisms. Intracellular Ca^2+^ concentration can be increased via Ca^2+^ cycling by the activity of SERCA and its regulator, sarcolipin, a mechanism for NST in skeletal muscle. This Ca^2+^ cycle can influence thermogenesis by increasing UCP1 as well. The FCC, a mitochondrial-localized futile creatine cycle involving creatine phosphorylation by CKB (creatine kinase B), and phosphocreatine hydrolysis represent other key UCP1-independent thermogenic mechanisms in classical BAT. In the figure, the blue arrow indicates a receptor-triggered signaling cascade leading to UCP1-dependent thermogenic induction, the orange arrow indicates a signaling cascade leading to UCP1-independent thermogenic induction, the dashed-line arrows indicate receptors or pathways associated with the central nervous system (CNS), and the dotted-line arrows indicate receptors linked to multiple thermogenic pathways.

Various pathways have been proposed to stimulate thermogenesis, which may be dependent and independent of UCP1. The best-known and most extensively studied pathway for activating UCP1 is the cAMP–PKA pathway. This pathway is triggered by β-adrenergic receptor stimulation. Common triggers include physiological stimuli (cold exposure, exercise) and certain pharmacological or dietary factors. This pathway represents the primary canonical route of browning. Other pathways, including cGMP-AKT, AMPK, mTOR, TGF-β/BMP, TRP channels, and Wnt/NF-κΒ/Notch/Hedgehog, have also been recognized for their roles in inducing thermogenesis (Figure 1). Overall, the pathways listed above form a systemic regulatory network that operates in an integrated and coordinated manner. cGMP-AKT and AMPK stimulate biogenesis of mitochondria and overall thermogenic gene expression. Downstream of these pathways, mTOR controls cell growth and energy metabolism. TGF-β/BMP and Wnt/NF-κB/Notch/Hedgehog crosstalk commands adipocyte maturation and plasticity, directly impacting the thermogenic capacity of brown and beige fat. TRP channels are multimodal sensors that can be activated by both mechanical and chemical stimuli (for example, changes in temperature, mechanical stress on cell membranes), leading to thermogenesis. This integrated signaling network allows brown and beige fat to react dynamically to external stimuli and undergo thermogenesis. Some of these above-mentioned pathways may overlap downstream with components of the β-receptor pathway, but also independently promote EE and thermogenesis. There are different receptors other than β that are involved in inducing thermogenesis through these pathways. Receptors such as GPR, TGF-β, GLP-1, NPR, TGR5, FXR, BMPR, SIRT, and DACRA regulate thermogenesis, along with hormones such as thyroid hormones, growth hormones, Estrogen, leptin, and irisin (Figure 1 and Figure 2). Additionally, the activation of transcription factors (PPARγ, FGF21, irisin) and the release of specific cytokines in adipocytes can induce thermogenesis (Figure 1). Thermogenesis can also occur independently of UCP1. In skeletal muscle and beige adipocytes, this is primarily driven by futile calcium cycling mediated by SERCA. Two distinct mechanisms operate: sarcolipin (SLN) enhances SERCA-mediated ATP hydrolysis via calcium slippage, whereas ryanodine receptor (RyR)-mediated calcium leak drives continuous reuptake by SERCA [35]. Both mechanisms ultimately dissipate energy as heat without mechanical work [35]. In classical BAT, Ca^2+^ cycling acts as an upstream regulator, enhancing UCP1-mediated thermogenesis [36]. Mitochondrial calcium uniporter (MCU) recruits UCP1 through the MCU essential regulator to increase mitochondrial calcium uptake to accelerate the TCA cycle and supply more protons to promote uncoupled respiration [36]. So, the action of Ca^2+^ cycling depends on the specific cellular context and the type of AT involved. UCP1-dependent thermogenesis is more prevalent in young, healthy adults, whereas futile Ca^2+^ cycling is activated mainly when UCP1 signaling is compromised, such as in thermoneutral states, aging, and obesity. As beige fat and skeletal muscle have far greater mass than classical BAT and remain metabolically active in obese individuals, UCP1-independent mechanisms (e.g., Ca^2+^ cycling) are promising therapeutic targets for combating obesity in humans.

Recent evidence suggests that the anti-obesity and anti-diabetic activities of FGF21 are independent of UCP1 [37]. Additionally, mitochondrial proton uncouplers such as 2,4-dinitrophenol (DNP), lactate, and salsalate can induce UCP1-independent thermogenesis by dissipating the proton gradient, yet their clinical use is severely limited by safety and toxicity concerns [38,39,40]. DNP, a potent mitochondrial uncoupler used for weight loss in the 1930s, markedly increases EE without reducing food intake, resulting in weight loss and improved glucose tolerance in rodents. However, its clinical use was abandoned due to severe toxicity and fatal hyperthermia [38]. However, chronic usage has been associated with persistent side effects in the liver, kidneys, and nervous system. Also, due to the narrow margin between effective and toxic doses, DNP is not considered safe for weight loss [41]. Salsalate uncouples mitochondria, activates skeletal muscle and BAT thermogenesis, protects against metabolic dysfunction due to a high-fat diet (HFD), and improves glucose homeostasis [39,40]. Furthermore, a growing body of literature suggests that BAT itself secretes metabolism-regulating factors, including peptide and non-peptide factors, which serve as signaling molecules for inter-organ or inter-tissue crosstalk (Figure 1) [42]. Thus, these secreted factors may also serve as potential therapeutic agents to combat obesity and other metabolic disorders. All these thermogenic pathways can be regulated not only by external stimuli but also by internal organ crosstalk through various pharmacological and non-pharmacological approaches.

## 5. Pharmacological Interventions to Induce Thermogenesis

Pharmacological induction of thermogenesis uses small molecules, peptides, and natural compounds to activate brown and beige AT, thereby increasing EE. The primary objective of anti-obesity pharmacotherapy is to shift the energy balance by either reducing caloric intake or increasing EE through thermogenesis. While initial efforts focused on the canonical sympathetic nervous system (SNS), current research is highly focused on leveraging alternative, non-adrenergic signaling pathways to improve clinical efficacy and safety. Although a few researchers are still exploring selective β_3_ (e.g., mirabegron, CL316,243) and β_2_ agonists (e.g., formoterol), most investigators now favor alternative approaches due to their limited efficacy and off-target effects in humans [6,9,43]. A wide range of natural and synthetic compounds are under investigation that activate thermogenesis either indirectly or by completely bypassing adrenergic receptors. Diverse natural (e.g., berberine, capsaicin, resveratrol) and synthetic (e.g., liraglutide, semaglutide) compounds enhance EE and thermogenesis primarily via AMPK-mediated PGC1α/UCP1 induction (Table 1). Beyond adrenergic and thermogenic pathways, other anti-obesity strategies under investigation include amylin analogs, leptin analogs, GLP-1 analogs, melanocortin-4 receptor (MC4R) agonists, oxyntomodulin, neuropeptide Y antagonists, cannabinoid CB1 receptor blockers, MetAP2 inhibitors (e.g., beloranib), lipase inhibitors, and anti-obesity vaccines [44]. Functional foods rich in soy- or marine-derived peptides are also widely consumed for their modest lipid-lowering effects [45]. Finally, exercise mimetics, such as the PPARδ agonist GW501516 and β-aminoisobutyric acid (BAIBA), aim to recapitulate the metabolic benefits of physical activity, although their clinical efficacy remains debated [46]. In the following sections, we review both canonical adrenergic approaches and emerging non-adrenergic pharmacological interventions that show considerable promise for safer and more effective obesity treatment.

### 5.1. Adrenergic Interventions (Canonical Pathways)

Adrenergic interventions involve stimulating the adrenergic receptors to induce thermogenesis. The thermogenic response to norepinephrine (NE) involves two basic pathways: one mediated via β-adrenergic receptors (βAR), which increases cAMP, and the other via α-adrenergic receptors (αAR), which increases cytosolic Ca^2+^ levels [71]. To date, nine different AR genes have been identified in the human genome, which encode nine different ARs that can be divided into two major groups (α and β) [72]. Although all these receptors are involved in thermogenesis, their degree of impact is still under investigation.

#### 5.1.1. Beta (β) Agonists: The Foundation of Thermogenic Therapy

β-adrenergic receptors (βARs) play a key role in regulating the SNS, mediating the effects of neurotransmitters such as epinephrine and NE. Numerous studies have demonstrated that the infusion of catecholamines, such as NE and epinephrine, increases EE, lipid oxidation, and lipolysis. All three subtypes of β-adrenergic receptors associated with thermogenesis are G-protein-coupled receptors (GPCRs). However, the functions and significance of the individual subtypes in the context of thermogenesis differ and remain under study. While β1ARs are more expressed in the heart and kidneys, β2ARs are expressed across the lungs, blood vessels, and uterus, and most extensively in adipocytes [6]. β3ARs are primarily expressed in the BAT, beige AT, and urinary bladder, with minimal expression in the smooth muscle of the heart, colon, and GIT, depending on the species [6,72]. The stimulation of β3ARs induces lipolysis in WAT and facilitates NST in BAT and beige AT [43,72]. Moreover, mutations in the β3AR gene have been correlated with insulin resistance (IR), increased risk for obesity and diabetes, and nonalcoholic fatty liver disease in obese individuals [6]. During cold exposure, exercise, and certain other stimuli, NE is released from the SNS and primarily binds to β3AR to trigger adrenergic signaling [73]. When coupled with Gs, β3AR activates adenyl cyclase (AC), leading to the production of cAMP and subsequent activation of protein kinase A (PKA) (Figure 2). PKA then targets several downstream effectors, including p38, CREB, and hormone-sensitive lipase (HSL), which promote thermogenesis by stimulating thermogenic gene expression in BAT and/or facilitating substrate mobilization to support thermogenic processes [73].

In rats and mice, β3-adrenergic stimulation significantly increases EE and lipid utilization [43]. However, the rat β3 adrenoceptor differs from the human β3AR at the genetic, structural, pharmacological, and functional levels. Consequently, the specific β3AR agonists used in rats have demonstrated weak agonism in humans. Adult human BAT and its adipocytes express β3AR mRNA, but at lower levels compared to rodents [8,74]. However, activation of β3AR using the selective agonist mirabegron enhances human BAT glucose uptake and metabolic activity, increases whole-body EE, and boosts plasma free fatty acid (FFA) [6,9]. In addition, obese subjects undergoing chronic mirabegron treatment showed increased UCP1 expression and phosphorylation of HSL on serine 660 in scWAT [75]. It should be emphasized that, in these studies, higher doses of mirabegron were used to activate β3 and increase BAT activity [6,9,75]. At high doses, mirabegron was unable to maintain blood pressure (BP) and heart rate, indicating a spillover to β1 and β2 receptors [76]. Moreover, mirabegron is approved only for overactive bladder; at this standard dose (50 mg/day), weight loss is minimal or absent [76]. So, mirabegron may be comparably more selective for β3 than other β-selective agonists, but high doses of mirabegron have compromised selectivity for β3. Fat-selective β3AR agonists may be a useful approach to mitigate side effects. Likewise, a novel adrenergic agonist, ATR-127, has been proven to be capable of targeting only skeletal muscle and BAT in mice [77]. However, a human study can be conducted to assess the translation of the effect in humans. So, to date, Mirabegron remains the most extensively studied and best-characterized pharmacological activator of human BAT. Future studies should focus on strategies to reduce the effective dose required for mirabegron-mediated thermogenic activation, including tissue-targeted delivery, optimized dosing regimens, and combination approaches that enhance thermogenic sensitivity while minimizing cardiovascular side effects. In addition, other β subtypes and noncanonical approaches should be explored.

Growing evidence now highlights the predominant role of the β2-adrenergic receptor, rather than β3 or β1, in human BAT activation, EE, and lipolysis in WAT. Functional studies have consistently shown that β2AR stimulation is the dominant trigger of human BAT thermogenic responses [8,74]. A significant association was found between a functional SNP and β2, but not β1 and β3, using fluorodeoxyglucose-positron emission tomography and computed tomography (FDG-PET/CT) [74]. Pharmacological stimulation and inhibition of the β2AR, along with the knockdown of β1, β2, or β3 in human BAT, collectively confirm that BAT lipolysis and thermogenesis transpire through β2AR signaling in humans [8]. Different β2 adrenergic receptor agonists, such as salbutamol and terbutaline, have been found to increase basal metabolic rate (BMR) and reduce body mass [78]. Formoterol, another selective agonist, has been found to be effective in boosting EE and lipid utilization [47]. Therefore, β2-receptor agonists can be targeted in conjunction with β3 agonists for anti-obesity treatment. However, β2ARs are not limited to AT; they are also present in multiple organs. So, an AT-selective β2 agonist, as well as both selective β2 and β3 agonists, may be capable of minimizing these side effects. Although β agonists can effectively activate BAT, their clinical utility is limited by off-target cardiovascular side effects.

The role of β1 in BAT thermogenesis is unclear and controversial. For a long time, β1 was believed to play a permissive or supportive role rather than serve as a primary thermogenic driver; this concept has been largely challenged by recent research in animals and humans [7,79]. β1 stimulation by dobutamine has been found to enhance UCP1 expression and stimulate glycerol release in human BAT [79]. The following study used human BAT biopsies and reported that β1 adrenoceptors are the primary mediators of thermogenesis in this tissue [79]. In contrast, Dumont et al. found that administration of the β1-antagonist bisoprolol with mirabegron reduced adverse cardiac side effects while facilitating thermogenic action in human BAT [7]. Although the evidence indicates the potential of β1AR in future anti-obesity therapy, without a direct assessment of whole-body EE and changes in lipid content, it remains inconclusive. Further research is required to determine whether it plays a supporting role or acts as a key thermogenic driver.

The relative contributions of β1, β2, and β3 adrenoceptors to human BAT thermogenesis might still be debated, but most researchers agree that β2 receptors play a major role, and in adults, a dominant role. However, it is undoubtedly true that all β subtypes have the potential to play a crucial role in combating obesity through thermogenesis, and each subtype comes with its own challenges and prospects, sometimes making it a debated approach for tackling obesity. Therefore, exploring other noncanonical pathways has garnered interest from researchers.

#### 5.1.2. Alpha (α) Agonists and Sympathetic Regulation

α-adrenergic receptors (αARs) are GPCRs that play an essential role in the regulation of blood pressure. They are divided into two main types. α1ARs are found in smooth muscle cells throughout multiple organs in the body, while α2ARs are more specifically found in the CNS. The increased density of α1ARs is associated with the activated state of BAT, and a positive effect of α1AR on thermogenesis in the BAT of rats has been proven [80]. The thermogenic action of α1AR can be both UCP1-dependent and UCP1-independent. UCP1-independent thermogenesis occurs through Ca^2+^ cycling, where an increase in cytosolic Ca^2+^ has been observed due to the activation or stimulation of α1ARs, whereas in UCP1-dependent thermogenesis, elevation of cAMP has been found [69,71]. Both α1 and α2 receptors play a role in facilitating BAT thermogenesis. In a study, Phenylephrine and the βAR agonist CGP 12177A individually increased oxygen consumption, while no synergistic action was observed when they were used in combination [80]. However, a selective α1 agonist, cirazoline, was added to forskolin, and it was found to have a potentiated thermogenic effect on the level of NE [81]. This suggests that rather than acting in isolation, α1ARs act in a co-dependent manner alongside β receptors.

On the other hand, α2ARs have been shown to inhibit thermogenesis by reducing sympathetic outflow to BAT and skeletal muscle [82]. Nano-injection of the α2AR agonist, clonidine (1.2 nmol), into the rostral raphe pallidus area (rRPa) inhibited BAT sympathetic nerve activity (SNA) and BAT thermogenesis [82]. Although little is known about the safety and effectiveness of α1 and α2 agonists in inducing thermogenesis in humans, they can be used as co-therapy with β agonists to modulate their efficacy, reducing side effects.

Adrenergic pathways have long dominated anti-obesity drug discovery. However, their poor specificity, cardiovascular risks, and limited success in humans require a change. The following sections explore non-adrenergic pathways that promise safer profiles and sustained metabolic benefits.

### 5.2. Non-Adrenergic Interventions (Novel Therapeutic Targets)

The complexity of thermogenic regulation necessitates the exploration of receptor families and signaling molecules that are independent of catecholamine signaling. These targets offer more specific, tissue-selective, and potentially safer approaches to promoting browning and activating thermogenesis. Non-adrenergic interventions constitute a noncanonical form of thermogenesis that can be UCP1-dependent and/or UCP1-independent. Their purpose is to activate thermogenic genes and proteins by bypassing adrenergic receptors, instead activating other transmembrane or nuclear receptors that target transcription factors in downstream pathways. Transmembrane receptors, including GPR3, TGR5, NPRs, GLP-1R, insulin receptors (IRs), and thyroid hormone receptors (THRs), as well as nuclear receptors such as estrogen receptors (ERs) and PPARγ receptors, have been shown to induce thermogenesis in BAT and scWAT. Hormones such as irisin and FGF21 also facilitate the expression of genes related to heat production. These pathways offer promising options for increasing EE when traditional pathways are compromised or absent.

#### 5.2.1. Peptides and Hormones in Crosstalk

##### Glucagon-like Peptide-1 Receptor Agonists (GLP-1RA) and Glucose-Dependent Insulinotropic Polypeptide Receptor Agonists (GIPRA)

GLP-1 is an incretin hormone secreted by intestinal L-cells in response to nutrient ingestion. It works in several ways: (i) it helps to stimulate the release of insulin, (ii) it inhibits the secretion of glucagon, (iii) it boosts insulin sensitivity in skeletal muscle, and (iv) it reduces the production of glucose in the liver. The metabolic effects of glucagon are mediated through GLP-1R, which is predominantly located in the liver, although it is also found in the brain, heart, kidneys, GIT, and AT. Several studies have demonstrated that weight loss and T2DM can be managed by administering GLP-1RA in humans and rodents [83,84,85]. GLP-1RAs function by suppressing appetite, slowing gastric emptying, and increasing feelings of fullness, collectively leading to reduced calorie intake [83]. GLP-1R has remained the most prevalent target for anti-obesity treatment for researchers and pharmaceutical companies over the last few decades. Wegovy (semaglutide) is widely used as an anti-obesity medicine. GLP-1R agonists increase EE via central (VMH-BAT) and peripheral mechanisms, though their contribution in BAT in humans requires clarification. However, multiple pieces of evidence indicate that GLP-1R agonists increase thermogenesis and EE [83]. GLP-1RA also improves the lipid profile by reducing TG content [85]. Moreover, they improve AT function by reducing inflammation [84]. GLP-1RA also helps to redistribute vWAT to scWAT [83]. Moreover, GLP-1RA has been found to stimulate the secretion of FGF21, a member of the FGF superfamily with various metabolic functions [85]. GLP-1RA is also found to increase leptin signaling, and leptin acts on the hypothalamus to reduce appetite and EE [83]. A detailed discussion of FGF21’s role and leptin’s role in regulating thermogenesis is provided in this section.

In addition, proglucagon-derived peptide-knockout mice (GcgKO) show a greater drop in body temperature and increased oxygen consumption after cold exposure compared to wild-type mice [86]. Gcg KO mice also display lower levels of thermogenic markers, including UCP1, DIO2, PPARγ, and PGC1α, in BAT during cold conditions, with reduced UCP1 levels in ambient conditions [86]. Recently, novel hypothalamic pathways have been identified as potential targets for GLP-1R. For instance, administering liraglutide, a long-acting analog, via central routes activates GLP-1 receptors in the ventromedial nucleus of the hypothalamus (VMH) [87]. This activation leads to the inhibition of AMPK, resulting in sympathetic-induced thermogenic activation in BAT, induction of browning in scWAT, and a reduction in food consumption [48,49].

The glucose-dependent insulinotropic polypeptide receptor (GIPR), another receptor for incretin hormones, plays a key role in T2DM and obesity when used as a co-agonist with GLP-1RA. GIPR activation activates the SERCA-mediated Ca^2+^ futile cycle, inducing EE in WAT mice and leading to weight loss [85]. So, GLP-1RAs are currently employed as anti-obesity therapies; however, the potential contribution of thermogenesis to fat needs to be explored. Currently, other than liraglutide and semaglutide, there are few studies on other GLP-1RAs, especially on obese people without diabetes. In addition, long-term use of GLP-1RAs may carry a high risk of pancreatitis, gallbladder diseases, and hyperthyroidism [85,88]. At the same time, due to the high cost of GLP-1RAs, many patients cannot afford them in the long term, and discontinuation of the therapy causes reinitiation of even more fat deposition [89]. Meanwhile, research on dual (GLP-1R/GIPR) and triple (GLP-1R/GIPR/GCGR) agonists indicates that they represent a promising obesity-specific therapy.

##### Natriuretic Peptides

Natriuretic peptides (NPs) are endogenous hormones secreted by the myocardium in response to myocardial distension and increased loading conditions. Already, three mammalian NPs have been identified and characterized, which are atrial natriuretic peptide (ANP), B-type natriuretic peptide (BNP), and C-type natriuretic peptide (CNP). Beyond their classic roles in renal and cardiovascular function, natriuretic peptides (NPs) contribute to energy homeostasis, glucose regulation, and thermogenesis through abundant NP receptors in AT [90,91]. Recent studies show that ANP and BNP promote TG lipolysis, mitochondrial uncoupling, and AT browning, thereby enhancing thermogenesis and improving insulin sensitivity [90,91]. The two primary receptors are cardiac natriuretic peptide receptor A (NPRA) and natriuretic peptide receptor C (NPRC). The binding of NPs to NPRA activates intracellular guanylyl cyclase (GC) activity, enhances cGMP production, and initiates the PKG-dependent signaling cascade [92]. Simultaneously, PKG triggers the activation of p38 MAPK, leading to the initiation of the thermogenic program in BAT through the PGC1α-UCP1 pathway (Figure 2) [92]. Since ANPs via PKG have been found to phosphorylate the same targets in adipocytes as β-agonists do through PKA, these two systems seem to share a common mechanism for performing lipolysis. So, it activates thermogenesis using the same pathway, bypassing βAR activation. Administering BNP to mice has also been found to significantly increase UCP1 and PGC1α in WAT and BAT, leading to enhanced respiration and EE [92]. Therefore, ANP and BNP may serve as practical tools for combating obesity. On the other hand, when NPs interact with NPRC, which lacks an intracellular GC domain, they trigger the internalization and degradation of the peptides, which is crucial for regulating the pool of available NPs and activating target cells [93]. Deleting NPRC specifically in AT enhances NP signaling and protects against diet-induced obesity and IR [93]. Higher levels of brown adipocyte marker genes and increased thermogenic activity have been identified in NPRC knockout (KO) mice [93].

Both ANP and BNP are available for clinical use. The human recombinant ANP (carperitide) has received approval in Japan for the treatment of acute heart failure and is currently undergoing clinical development in the United States [94]. In 2001, the U.S. approved human recombinant BNP (nesiritide) [94]. Urodilatin (ularitide) is now in clinical development in both Europe and the U.S. Designer NPs may be an alternative solution to combat obesity [94]. Consequently, the development of NPR-specific agonists could present innovative strategies to activate BAT and enhance metabolic health. Since NP-induced lipolysis manifests more prominently in primates than in rodents, further research is necessary to elucidate human-specific mechanisms and refine pharmacological approaches [95]. Combining NP-based therapies with low-dose selective β3 agonists may exhibit synergistic efficacy, as both mechanisms increase cAMP activity. This approach might help optimize therapeutic efficacy while minimizing adverse effects. Thus, NPs represent a promising adjunct or alternative to adrenergic agonists in forthcoming anti-obesity treatments.

##### Fibroblast Growth Factor (FGF21)

FGF21, a member of the large FGF cytokine family, is a multifunctional endocrine growth factor involved in metabolic networks. It is secreted primarily from the liver and AT in response to nutrition-sensitive transcriptional factors and/or intracellular stress, such as protein malnutrition. It acts both locally and via interorgan crosstalk. Although the exact mechanism remains unclear, FGF21 plays multiple metabolic roles through its endocrine, autocrine, and paracrine actions [96]. In hepatic tissue, it lowers TG levels by decreasing lipid synthesis through de novo lipogenesis and increasing fatty acid oxidation (FAO) in the liver [96]. In AT, FGF21 enhances glucose uptake, promotes mitochondrial biogenesis, elevates oxidative metabolism, and stimulates the secretion of adiponectin [96]. So, FGF21 is a promising therapeutic target for the treatment of obesity, CVD, and fatty liver disease.

FGF21 functions through a receptor complex made up of fibroblast growth factor receptor 1 (FGFR1) and the co-receptor β-Klotho. This pairing is crucial for signal transduction, which triggers downstream signaling molecules like FRS2α and ERK1/2 [96]. Targeted genetic models lacking β-Klotho in AT demonstrate that direct signaling to adipocytes is necessary for the acute insulin-sensitizing effects of FGF21, though not for its chronic impacts on EE and weight loss [37]. FGF21 is crucial for adaptive thermogenesis, with cold and β-adrenergic stimulation boosting its expression in AT (especially BAT) to promote thermogenic genes like UCP1 [52,97]. Meng et al. demonstrated the paracrine action of FGF21, showing that mir-182-5p, a microRNA expressed in AT that regulates thermogenic genes and EE in mice, increases FGF21 expression and secretion [98]. FGF21 then stimulates acetylcholine secretion in M2 macrophages, thereby enhancing PKA signaling and ultimately promoting UCP1 expression in AT [98]. This process mediates the interaction between adipocytes and AT-resistant macrophages, stimulating browning in scWAT [98]. They also demonstrated that overexpression of mir-182-5p in the scWAT fat pad of mice confers resistance to obesity and its metabolic effects [98]. Some evidence supports the involvement of FGF2 and FGF9 in the upregulation of thermogenesis [99]. However, FGF21 has sparked considerable interest in the biopharmaceutical development of FGF21-based therapies. Although most clinical trials have focused on resolving metabolic-dysfunction-associated steatohepatitis (MASH), its potent antidiabetic and anti-obesity effects still need to be translated to humans [100]. FGF21 analogs and mimetics, such as LY2405319, PF-05231023, pegbelfermin, and pegozafermin, showed mild to no effects on weight loss, glycemic control, and MASH [100]. FGF21, in combination with GLP-1RA, is also under clinical trial to assess its efficacy in T2DM and obesity. In the future, more clinical trials should be conducted focusing on obesity and its associated metabolic disorders.

##### Irisin

Irisin, a small adipo-myokine polypeptide hormone, is produced from the cleavage of fibronectin type III domain-containing 5 (FNDC5). It is mainly secreted by skeletal muscle and AT. Its production is stimulated by physical activity through the activation of PPARγ, PGC1α, and FNDC5, thereby helping to convert white fat into brown fat [101]. Circulating irisin levels have been observed to be higher in physically active individuals, while sedentary individuals show lower levels [101]. Moreover, irisin demonstrated similar effects to exercise in improving specific parameters related to metabolic disorders, including adipokines, insulin, ghrelin, and BMP4 [102]. Subjecting animals to swimming exercise resulted in a significant increase in serum irisin levels and a reduction in fat content [103]. The mechanism by which exercise can improve metabolic health by inducing BAT thermogenesis is described in Section 6.3. So, irisin and its analogs may mimic some metabolic benefits of exercise. However, further studies are needed to support such a claim.

Functionally, irisin exerts various metabolic effects via multiple pathways, primarily the MAPK, PI3K/AKT, p38-ERK, and JAK/STAT signaling pathways [101,103]. In mice, irisin and FNDC5 support adipose–bone connectivity by PRDM16 induction, which is important both to induce browning in scWAT and for the development of skeletal muscle [104]. Murine preadipocytes exposed to FNDC5/irisin show increased UCP1 and thermogenic genes, while classical brown adipocytes do not respond, indicating depot-specific effects [101]. Wu et al. identified a distinct progenitor population within WAT adipocytes capable of giving rise to beige adipocytes, which is not present in classical BAT [22]. Notably, CD137-high preadipocytes exhibited a markedly stronger response to irisin and FNDC5-induced browning than cells low in CD137 [22]. Additional studies confirmed that irisin facilitates scWAT browning and enhances metabolic health through the p38 MAPK and ERK signaling pathways, as well as other supporting mechanisms [22]. However, human studies show highly variable and often contradictory effects of irisin on adipose browning. Raschke et al. found that neither recombinant FNDC5 nor irisin induced browning in primary human subcutaneous preadipocytes [105]. They further showed that high CD137 expression in human subcutaneous AT did not correlate with FNDC5/irisin-induced browning, contradicting the findings reported by Wu et al. [22,105]. The beneficial effects of irisin in obesity and other metabolic disorders in humans remain to be explored.

##### Leptin

Leptin is a hormone primarily involved in managing energy balance by suppressing appetite and energy homeostasis. It is also vital for temperature regulation. Research has shown that leptin can regulate thermogenic capacity by modulating UCP1 expression in BAT [106,107,108]. For instance, mice that lack leptin exhibit excessive eating, severe obesity, and an inability to maintain body temperature in cold conditions [108]. Leptin supplementation in these mice improved body temperature regulation without altering EE, suggesting it aids thermoregulation by decreasing heat loss in addition to stimulating thermogenesis [106,107,108]. Leptin injections have been found to lower body fat and food intake while increasing core temperature, metabolic rate, and the activity of several thermogenic and mitochondrial markers, as well as cytochrome c oxidase in the liver [108]. Chronic leptin treatment increases UCP1 expression and mNRG4 expression in BAT, promoting thermogenesis through direct and sympathetic intervention in mice [109].

Leptin may also support thermoregulation by affecting SNA, which enhances fat breakdown in WAT and boosts thermogenic gene expression in BAT [110]. Direct injection of leptin into the brain’s ventricular system also increased energy output and impacted the sympathetic activity in BAT [111]. Leptin’s effects on EE and body temperature are believed to involve the hypothalamus, especially the DMH [111]. Nevertheless, the precise mechanisms through which leptin activates the SNS and modulates thermogenic responses remain under investigation. Moreover, most leptin analogs are used in cancer therapy. Recently, several clinical trials have been conducted to explore the impact of metrileptin, a leptin analog, on treating obesity [112]. Future studies can be designed that combine GLP-1RA or GH with leptin analogs to explore their additional/synergistic therapeutic effects in obesity.

#### 5.2.2. G-Protein-Coupled Receptor (GPCRs)

##### G-Protein-Coupled Receptor 3 (GPR3)

GPR3 is a member of the GPCR subfamily and an orphan receptor with a high constitutive G-coupling activity level. Although it is widely expressed across brain regions and functions centrally, it has also been detected in BAT and WAT [113]. A recent study has found that a noncanonical lipolytic signal directly stimulates GPR3 transcription [113]. The research indicates that the N-terminus of GPR3 possesses an inherent capability to signal proficiently through G coupling, independent of an external stimulatory ligand. This increase in GPR3 expression can significantly enhance cAMP-mediated adipose thermogenesis [113]. Cold-induced GPR3 expression via a noncanonical lipolytic signal triggers the thermogenic program in brown and beige adipocytes in both mouse models of metabolic disease and humans [113]. A targeted qPCR array to investigate 44 G-coupled receptors in mice proved that GPR3 was the most profoundly cold-induced receptor in scWAT and BAT [113]. In addition, the β1AR, β2AR, and β3AR were genetically ablated in mice to confirm that GPR3 signaling was independent of adrenergic agonism [113]. In vitro research on human brown adipocytes showed that knocking down GPR3 significantly decreased UCP1 expression [113].

However, the mechanisms by which GPR3 facilitates ligand-independent constitutive G coupling remain unclear. Using mass spectrometry (MS) analysis and functional assays, oleic acid (OA) has been identified as a ligand for GPR3, highlighting key aspects of GPR3 ligand binding [114]. Additionally, Yangjie Xiong and colleagues demonstrated that cold exposure triggers the release of OA, which activates GPR3 in mice [114]. Mice lacking GPR3 were found to put on weight due to impaired thermogenic function of BAT and face challenges in maintaining thermal homeostasis in acute cold exposure [115]. Thus, GPR3R provides a cAMP-mediated thermogenic pathway that is independent of AR signaling, thereby identifying it as a noncanonical regulator. However, activating GPR3 may compensate for the efficacy lag due to low-dose selective AR agonists. In addition, the integral activity of GPR3 may present both opportunities and risks. Its constitutive activity enables ligand-independent signaling but complicates pharmacological control. Additionally, most insights are from rodent models, and there is very limited evidence available on the support of GPR3 expression in brown and beige AT. GPR3 agonists/modulators are promising next-generation thermogenic targets for obesity and metabolic disease, as they circumvent the AR desensitization and cardiovascular side effects of β-adrenergic agonists.

##### TGR5 (Bile Acid Receptor)

Bile acid (BA), one of the main components of bile, is synthesized in the liver, and its excretion is dependent on dietary intake. Besides its regular functions, such as aiding in the absorption of nutrients and regulating the growth of gut microbes, it can also regulate glucose homeostasis and energy levels in humans and mice [116,117,118]. BA acts in various tissues and organs via receptor-mediated mechanisms. Farnesoid X receptor (FXR, NR1H4) and Takeda G-protein-coupled receptor 5 (TGR5/GPBAR1) activation sends signals to downstream genes, regulating energy homeostasis in the body [116]. The activation of TGR5 promotes thermogenesis and EE in muscle and BAT through activation of thermogenic genes such as DIO2 and UCP1 [116]. In addition, bile acid promotes glucagon-like peptide-1 (GLP-1) release through TGR5 and cAMP activity [118].

TGR5 agonists increase cAMP levels, thereby enhancing PKA activity, which significantly enhances DIO2 expression, improves T4 conversion, increases T3 levels, and enhances energy consumption and fat metabolism (Figure 2) [116]. The TGR5 agonist INT-777 can enhance the phosphorylation of CREB via the cAMP-PKA pathway [119]. CREB is fundamental in regulating the transcription and expression of the mitochondrial thermogenic genes PPARγ and PGC1α [120]. Therefore, TGR5 agonists can activate thermogenesis via the cAMP-PKA pathway, sidestepping β. TGR5 regulates the futile creatine cycle (FCC) for UCP1-independent heat production by increasing mitochondrial creatine kinase 2 (CKMT2) expression, which promotes energy use in beige and brown AT [116]. TGR5 agonists also protect mice against alcohol-induced steatosis and liver injury by inducing BAT thermogenesis and reducing FFAs [117].

Recent studies have demonstrated that TGR5 is also expressed in the hypothalamus, where it plays a vital role in BA signaling and the regulation of obesity. Administering BAs or a TGR5 agonist has been shown to decrease body weight and fat mass by activating the SNS, thereby creating a negative energy balance [121]. Conversely, reducing TGR5 expression in the mediobasal hypothalamus has been shown to trigger the onset of obesity and worsen existing obesity by decreasing sympathetic activity [121]. In a recent study, Bruce et al. found that activating TGR5 in the brain lowered food intake by enhancing leptin-STAT3 signaling [122]. Therefore, TGR5R exhibits significant therapeutic potential in addressing obesity. However, compared with other organs, TGR5 expression in BAT and the brain is much lower. In addition, overexpression of TGR5 alleviates the possibility of myocardial ischemia/injury due to elevated ROS, stabilized mitochondrial membrane proteins, and reduced intracellular Ca^2+^ concentration [123]. So, it necessitates further study to establish if the appetite suppression is exclusively attributed to TGR5. Additionally, TGR5 stimulates GLP-1 secretion, which has promising results for weight loss. RO5527239, an experimental TGR5 agonist, has been found to elevate GLP-1 and polypeptide YY (PYY) hormone co-secretion from L cells in the gut [124]. So, TGR5 agonists can be beneficial for obesity treatment in a multimodal way and hold promise for future studies in humans to treat obesity and other metabolic disorders. There are many more TGR5 analogs that should be studied from the perspective of obesity treatment as well.

The function of the other bile acid receptor, FXR, in BAT is still unclear. According to some research, FXR activation counteracts thermogenesis triggered by TGR5. Yang et al. demonstrated that FXR expressed in newborn cholestatic Cyp2c70^-/-^ mice causes BAT whitening and impairs its development and function by upregulating UCP1 and DiO2 [125]. Fexaramine, an FXR agonist confined to the gut, has demonstrated the ability to promote browning and enhance energy utilization by BAT while remaining in the gut’s bloodstream [126]. The combination of TGR5 activation with FXR modulation or dual-acting agonists has the potential to enhance thermogenesis and improve energy balance. 7β-isopropylchenodeoxycholic acid, a dual potent FXR antagonist/TGR5 agonist, has already been found to be effective in improving glucose metabolism and increasing the secretion of GLP-1 in mice [127]. Emerging strategies, such as engineered BA analogs, nanocarriers, and AI-driven design, represent exciting efforts to develop targeted agents that may be advantageous in combating obesity.

#### 5.2.3. Sensory Inputs and Ion Channels

##### Transient Receptor Potential (TRP) Channels

**Figure 3:**
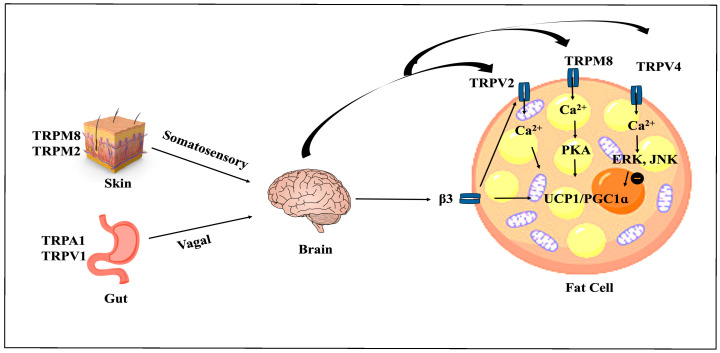
Mechanisms of thermogenesis induction in brown and beige adipocytes by sensory nerves expressing TRP channels. The figure illustrates how calcium influx mediated by TRPV1, TRPA1, TRPV2, TRPV4, TRPM2, and TRPM8 regulates thermogenic gene expression in adipocytes, leading to increased thermogenesis. Additionally, heightened SNA results in the release of NE from the SNS and the activation of β3 receptors in BAT. TRPV2 works synergistically with β3 to regulate PGC1α and UCP1, further enhancing thermogenesis. Conversely, TRPV4-mediated calcium influx negatively regulates thermogenic gene expression in adipocytes, thereby inhibiting thermogenesis. In the figure, the black arrow indicates different pathways followed by different TRP channels. In the fat cell, the yellow circles indicate lipid droplets, the purple ovals indicate mitochondria, and the orange circle indicates the nucleus.

Transient receptor potential (TRP) channels are ion channels that are permeable to non-selective cationic ions. To date, 28 different TRP channel genes have been identified based on primary amino acid sequences [128]. TRP channels are responsible for a range of sensory responses, including heat, cold, pain, stress, vision, and taste, and may be activated by a variety of stimuli [128]. TRPs distributed in the skin, fat, skeletal muscles, and brains of animals are closely related to activation of BAT thermogenesis [128]. A few of them are found in adipocytes or help adipocytes contribute to energy metabolism and inflammation in AT. These TRPs are thermosensitive: TRPV1, TRPV2, TRPV4, and TRPM2 are activated by warmth/heat (27–52 °C), and TRPA1 and TRPM8 are activated by cold (below 17 °C and 27 °C, respectively) [128]. In addition, TRP channels are activated by diverse physical (temperature, pressure, force) and chemical (pH, osmolarity, ligands) stimuli. This activation facilitates calcium influx, resulting in increased intracellular Ca^2+^ levels [128]. However, the mechanism is different for each channel (Figure 3).

The TRPV1 receptor, primarily known for detecting pain and temperature, is generally expressed in sensory neurons; it has been found throughout the body, including the gut, skin, and ATs. TRPV1 can be activated by capsaicin and capsinoids, which are selective agonists of the TRPV1 receptor. However, TRPV1 capsaicin was found to raise intracellular Ca^2+^ levels in 3T3-L1 adipocytes and in beige and brown fat of mice in a TRPV1-dependent manner, resulting in SIRT1 phosphorylation through AMPK activation [55]. It also improved the interaction between PPARγ and PRDM16, promoting WAT browning [55]. The activation of TRPV1 by capsaicin demonstrated a comparable effect in BAT, countering obesity by boosting metabolism and EE [55]. Capsinoids and capsaicin analogs have been found to boost BAT thermogenesis, enhancing EE and fat oxidation in overweight and obese mice [55].

TRV2 is expressed in multiple tissues, including lymphocytes, macrophages, and neurons, and is involved in regulating both the immune and nervous systems. However, TRPV2-deficient (TRPV2KO) adipocytes in mice exhibit reduced mRNA levels of several genes, including UCP1 and PG1α, which are also associated with mitochondrial oxidative metabolism [129]. Moreover, TRPV2 KO adipocytes showed reduced responses to the β-adrenergic agonist due to the lack of TRPV2-mediated calcium influx [129]. TRPV2 KO mice have shown increased fat deposition and body weight after consuming an HFD [129]. Very recently, Iwase et al. found that Chloramine-T (a methionine oxidant) activated TRPV2 at around body temperature and increased thermogenic gene expression in TRPV2-transfected HEK293T cells and mouse BAT [130]. So, TRPV2 could play a potential role in combating obesity in humans, though there are still no reports available on the expression of TRPV2 in human BAT.

The role of TRPV4 in energy metabolism remains unclear. Until a few years ago, it was known that TRPV4 negatively regulates thermogenesis by inhibiting PGC1α and UCP1 in mice. TRPV4 agonism has been shown to rapidly phosphorylate ERK1/2 and JNK1/2, which, in turn, inhibit activation of p38 MAPK, a key pathway that activates the downstream thermogenic cascade [131]. Both basal and NE-stimulated expression of PGC1α and UCP1 were downregulated due to the knockdown of TRPV4 in mouse BAT, and TRPV4-deficient mice exhibited enhanced muscle energy oxidation and resistance to HFD-induced obesity [131]. However, a few years ago, Zhang et al. found the opposite. They claimed that TRPV4 can directly promote WAT browning via the AKT pathway in mice [132]. Moreover, TRPV4 KO causes exacerbation of DIO due to impaired thermogenesis [132]. Although human data remain limited, TRPV4 shows strong preclinical promise as a novel therapeutic target for obesity and related metabolic disorders.

TRPM8 is a cold receptor that can be activated not only by cold temperatures but also by other cooling agents, such as menthol and eucalyptol. Among all the TRPs, TRM8 is the most extensively studied, shown to be involved in BAT thermogenesis [128]. TRPM8 activation can stimulate cold-sensing receptors, mimicking environmental cold exposure. Menthol, a cool-inducing agent, activates TRPM8, and TRPM8 activation leads to PKA activation, UCP1 upregulation, mitochondrial activation, and heat production [63,64]. Both topical and oral menthol stimulate BAT thermogenesis and WAT browning in normal and diet-induced obesity in mice [63,64]. Menthol promotes hyperthermia, resulting in reduced weight gain in rats [63]. Menthol in the diet significantly raised core body temperatures and locomotor activity in WT mice, but had no effect on TRPM8 KO and UCP1 KO mice [63]. Topical menthol application has been found to enhance gluconeogenic activity, whole body EE, and lipid utilization [63]. Despite menthol’s less significant effect in humans compared to animals, Rossata’s team found TRPM8 in WAT, where menthol induces browning properties (UCP1 expression, mitochondrial activation, and heat production) [133]. Further study of oral and topical menthol may reveal a new application of this compound in the treatment of obesity.

Moreover, other TRP channels, such as TRPA1, TRPC1, TRPC5, and TRPP3, have also been identified, showing potential to regulate BAT thermogenesis and mitochondrial respiration [134]. Loss of TRPC5 has been identified to cause obesity in humans [135]. So, TRP channel agonists act via different mechanisms to avert obesity. In addition, various dietary constituents such as, capsaicin, capsinoids, allicin, cumin, oleuropein, and menthol, have been found to show anti-obesity effects by activating TRP channels. So, combining these agonists might result in an additive or synergistic anti-obesity effect. A combination of capsaicin, menthol, and cinnamaldehyde was potentially able to induce EE and glucose uptake in mice [136]. Additionally, combination therapy will lead to individual dose minimization, ensuring fewer side effects and a less pungent taste of spices. Proper human studies of these combination therapies, keeping the dose reliability, adherence, and safety under consideration, might guide us toward the development of a superfood for preventing obesity. At present, such dietary combinations should be viewed as adjunctive and hypothesis-generating approaches, with primary outcomes clearly distinguished between thermogenic activation and appetite or behavioral modulation.

##### Other Ion Channels

Several ion channels (e.g., calcium and potassium) are intimately involved in thermogenesis and represent promising therapeutic targets for obesity treatment. Thermogenesis in skeletal muscle, BAT, and beige AT can be UCP1-dependent and independent. Calcium-cycling-mediated thermogenesis represents a key UCP1-independent mechanism of NST. In contrast, KCNK3, a two-pore-domain potassium channel, promotes lipid accumulation and suppresses thermogenesis via AMPK and AKT signaling [137]. KCNK3 acts as a brake on adrenergic activation in brown adipocytes by facilitating K^+^ efflux. It counteracts NE-induced depolarization, reducing activation of voltage-dependent Ca^2+^ channels [138]. This restriction of Ca^2+^ entry diminishes downstream adrenergic signaling, reduces lipolysis, and ultimately decreases thermogenic respiration [138]. AT-specific KCNK3 KO mice exhibit increased EE and resistance to obesity [138]. However, these findings need to be translated to human studies to confirm the KCNK3 channel as a potential target for obesity therapy.

In skeletal muscle, the ER/SR stores Ca^2+^ and continuously cycles it through SERCA-mediated uptake and RyR/IP3R-mediated release back to the cytosol, creating a futile Ca^2+^ cycle that consumes ATP and generates heat. SERCA usually hydrolyzes ATP to pump Ca^2+^ ions in the sarcoplasmic reticulum, and SLN, a transmembrane protein, potentially facilitates ATP hydrolysis by binding with SERCA. However, even though ATP is continuously hydrolyzed, Ca^2+^ leaks out, leading to the release of energy as heat [40]. The second mechanism is the futile cycling mechanism, wherein calcium ions continuously leak out through the RyRs and are pumped back in by SERCA, continuously expending ATP, but without causing contraction (in the case of skeletal muscle) [139]. Phosphorylation of RyR by PKA induces calcium leak through SR by destabilizing calstabin2 binding, thus driving a continuous cycle of calcium being pumped in, ATP being hydrolyzed, and heat generation [139]. As ATP synthesis primarily drives this mechanism, it occurs in a higher proportion in beige fat and skeletal muscle compared to brown fat, where ATP generation is a second priority. This SERCA uncoupling activates the AMPK/PGC1α signaling pathway, accelerating mitochondrial biogenesis and oxidative metabolism, thereby increasing UCP1 expression [140]. While most studies have centered on skeletal muscle, evidence indicates that SERCA activity in beige adipocytes is also important for UCP1-independent thermogenesis. SERCA2b is the isoform commonly found in beige AT that promotes heat generation through the Ca^2+^ cycle, helping to reduce insulin resistance and improve glucose tolerance [139,141]. Increased Ca^2+^ leakage represents another mechanism of heat production through the calcium cycle by increasing cytosolic Ca^2+^ concentration, the same mechanism we observe due to α-adrenergic stimulation. The UCP1-independent calcium cycle significantly boosts EE and prevents weight gain in mice [140]. When BAT is genetically or functionally impaired, skeletal muscle compensates by increasing SLN expression, raising mitochondrial content, and enhancing Ca^2+^ cycling, thus making muscle the primary thermogenic organ [140]. In traditional BAT, the futile creatine cycle (FCC) drives mostly ATP-linked UCP1-independent thermogenesis, accelerating ATP turnover through creatine phosphorylation and phosphocreatine hydrolysis [142]. Certain natural compounds, including capsaicin, epigallocatechin gallate (EGCG), baicalein, rosmarinic acid, and resveratrol, have been found to either facilitate or increase the expression of SERCA, which ultimately increases thermogenesis [54,143]. However, further research is required to develop these substances into effective anti-obesity therapies.

#### 5.2.4. Hormone Receptors and Small Molecules

##### Thyroid Hormones and Analogs

Thyroid hormones (THs) play a critical role in regulating metabolic and thermoregulatory processes in mammals and are known to regulate BMR in the human body as well. For decades, THs have been recognized as potent enhancers of obligatory thermogenesis, increasing energy expenditure and body temperature across multiple tissues, particularly skeletal muscle [144,145]. Hypothyroidism is often associated with increased fat deposition due to a marked reduction in BMR, thermogenesis, and mitochondrial function [145,146]. Recently, Roth et al. suggested that THs directly regulate Zfp423, an anti-thermogenic factor in beige AT, and that this regulation is essential for UCP1-dependent thermogenesis to boost EE in mice [147]. The primary mechanism by which THs work is the conversion of thyroxine (T4) to triiodothyronine (T3) with the participation of type 2 deiodinase (DIO2), the gene expressed in BAT (Figure 2) [145]. The SNS acts synergistically with THs to modulate BAT activity by increasing the expression of DIO2, expanding the availability of THs in the BAT [145]. T4 binding to TRs enhances the effects of β3AR stimulation on the activity of BAT. Moreover, T3 has a direct impact on browning that does not rely on the SNS, such as promoting mitochondrial autophagy by activating SIRT1 and reducing the activity of mTOR [148]. Moreover, free T4 and T3 are found to be positively associated with cold-induced thermogenesis (CIT) in euthyroid individuals [149]. These highlight the diverse ways THs influence metabolism and energy regulation.

The two primary nuclear THRs, TRα1 and TRβ, play a crucial role in TH function. They act as ligand-dependent transcription factors. TRα1 and TRβ receptors are expressed in different tissues, including BAT. TRβ regulates adaptive thermogenesis by influencing UCP1 mRNA expression [150]. The dependence of UCP1 expression in BAT on the TRβ isoform was subsequently confirmed in TRβ KO animals [145]. It has also been revealed that the T3 hormone and the selective TRβ agonist GC-1 induce browning in scWAT by stimulating UCP1 mRNA expression [151]. GC-1 can increase the metabolic rate both in vitro and in vivo, ensuring not only the induction of thermogenic gene expression but also the activation of adaptive thermogenesis [151]. Even maternal THs play a significant role in fetal development, and maternal TRβ activation in mice induces BAT thermogenesis in their offspring [152]. Limited information is available about the role of TRα1 in BAT thermogenesis and EE, although it is more significantly involved in maintaining body temperature [144]. Some researchers observed no change in UCP1 after knocking out TRβ, which might suggest a compensatory action of TRα. TRα signaling is vital for adrenergic responses, as its absence leads to a reduced reaction to BAT stimulation by NE [145]. However, hormone T3 can induce hyperthyroidism, which causes damage to bone structure and has harmful effects on the cardiac system due to binding with TRα [153]. Therefore, precisely selective TRβ agonists may be a viable option for anti-obesity drugs that activate thermogenesis. Both in vivo and in vitro studies prove that ZTA-261, a more selective and less toxic TRβ agonist, has potential for treating lipid-related disorders [153].

##### Estrogen Hormone and Analogs

Although primarily known as a female sex hormone, estrogen is also involved in various non-reproductive functions, including maintaining bone health, cardiovascular function, and energy homeostasis [154,155]. Estrogen deprivation has been associated with a higher risk of obesity and metabolic issues [154]. Estrogen receptors are found both in the membrane and in the nucleus of the ovaries, liver, and fat cells. The two main types of ERs are ERa and ERb. Obesity is linked to a marked reduction in the expression of both nuclear ER subtypes in AT, whereas weight loss results in upregulation [156]. A lower level of ERa mRNA expression was observed in the scWAT of obese patients, regardless of sex [156]. However, the function and role of the ER are sex-specific. Women suffer more due to hormonal fluctuation at different stages of their lives, such as puberty, labor, and menopause. A significant difference has been found between pre- and postmenopausal women [156]. This is because the ERa plays a stronger physiologic role in females than in men, and postmenopausal women are susceptible to obesity due to estrogen deprivation. In contrast, the activity of ERb is similar in both males and females.

So, it is certain that estrogen is an important regulator of obesity and metabolic syndrome both in humans and animals. However, the exact mechanism of action is still unclear. There are no available study that clarify the comparative expression of ERa and ERb in AT. Additionally, it is not certain that ER-induced weight loss is due to thermogenic activation. Research has shown that ERa in the VMH plays a crucial role in controlling spontaneous physical activity, EE, and thermogenesis, and knocking out ERa in the VMH leads to weight gain [155]. Estrogen, via Era, increases STAT3 signaling in the hypothalamus to reduce leptin resistance [157]. Tamoxifen, a selective estrogen receptor modulator historically used for treating breast cancer, has been found to increase leptin sensitivity in young animals [157]. Estrogen influences appetite control, reducing energy intake. However, the function of Tamoxifen is controversial because of its tissue-specific estrogen agonistic and antagonistic behavior, which restricts its future potential as an anti-obesity therapy.

An animal study confirmed that estrogen can induce thermogenic activity in BAT of female rats, whereas no change was observed in male rats [158]. A study by Lapid et al. found that selective ER deficiency boosts BAT differentiation, and that ER KO exhibits increased EE and enhanced glucose sensitivity due to increased TGF-β activity [159]. Therefore, estrogen may regulate fat deposition and breakdown through a multimodal approach, involving both central and peripheral mechanisms. The complexity of ER involvement in thermogenesis necessitates further research to inform a therapeutic approach. Not many estrogen analogs have been studied from an obesity treatment perspective whereby they target AT or central ERs. Further human studies of AT-selective estrogen receptor modulators or agonists could provide a novel therapeutic avenue for obesity.

##### Growth Hormone

Growth hormone (GH) regulates growth and metabolism by interacting with its specific receptor. Impaired GH signaling is associated with disrupted growth and a range of metabolic diseases, whether the deficiency is genetic or acquired. Lowering GHR activity in fat promotes a unique form of obesity with some protective metabolic features, despite an initial drop in cold resistance [160]. The absence of GHR in AT resulted in increased weight gain under HFD conditions. Bariatric surgery-induced changes in GH improved AT function [161]. Though the rise in GH correlated with a reduction in FFA and improved insulin sensitivity, it did not correlate with body weight [161].

GH promotes lipolysis and preserves lean mass in humans; consequently, GH deficiency increases body fat and reduces muscle mass in humans [162]. The same scenario has been observed in mice as well [163]. These shifts in body composition lower daily energy expenditure, as lean tissue is far more metabolically active than AT [160]. GH activates the MEK-ERK pathway, which is crucial for PPARγ inactivation and FSP27 reduction, thereby enhancing lipolysis and IR [162]. Additionally, GH treatment promotes the conversion of TH T4 to its active form, T3, thereby increasing metabolic activity. It also appears to increase appetite, which may lead to higher energy turnover.

Interestingly, GH has contrasting effects in the brain. During extended periods of food scarcity, GH can lower EE by activating certain neurons (AgRP neurons) that suppress thermogenesis and other energy-demanding functions [164,165]. Mice lacking GHR in these neurons exhibit a disruption in this energy-saving mechanism, resulting in higher EE during fasting [164]. This suggests that GH signals to the brain to conserve energy in times of nutrient shortage, a mechanism that is compromised when GHR is missing in specific brain areas [166]. Moreover, blocking GHR using specific antagonists can reverse this energy-conserving state, much like the hormone leptin [164].

In summary, GH influences metabolism through multiple mechanisms, enhancing energy utilization and thermogenesis in peripheral tissues while promoting EE in the CNS during starvation. These diverse effects underline GH’s pivotal role in balancing energy needs under different physiological conditions.

##### Sirtuins (SIRT)

Sirtuins are a family of highly conserved NAD+-dependent histone deacetylases involved in various biological processes, including insulin regulation, mitochondrial biogenesis, energy metabolism, bone development, and aging [167,168]. They play essential roles in AT remodeling in obesity, positioning them as potential targets for the treatment of obesity and metabolic disorders [169]. Humans have seven sirtuin homologs (SIRT1-7), each with distinct subcellular localization and substrate preferences [169]. Their expression varies across AT types and is influenced by diet and body fat levels [169]. Although all sirtuin homologs are involved in AT biology and lipid mobilization, SIRT1, 3, 5, and 6 are explicitly linked to thermogenesis. Both in vitro and in vivo studies suggest that SIRT1 plays a vital role in adipocyte differentiation, lipid mobilization, AT inflammation, and browning [169]. Upregulating SIRT1 decreases adipogenesis, increases lipolysis, and reduces proinflammatory responses [169,170]. It enhances AT browning by deacetylating PGC1α, suppressing PPARγ, activating AMPK signaling and FGF21, and inhibiting NF-κB signaling [169]. SIRT1 has also been proven to be involved in maintaining BAT capillarization [171].

SIRT3 preserves brown adipocyte identity and mitochondrial function by promoting differentiation via PGC1α, reducing lipid accumulation through AMPK–ULK1-mediated lipophagy, and suppressing inflammation by inhibiting the NLRP3 inflammasome [169]. SIRT3 deficiency causes browning to revert to white fat, characterized by increased lipid storage, decreased expression of UCP1 and perilipin-1, and an increased extracellular matrix, including collagen IV and VI [172].

SIRT5 regulates adipocyte metabolism by inhibiting differentiation and lipid buildup through AMPK activation and MAPK inhibition [169]. Its deficiency decreases FAO and may increase lipolysis [169]. SIRT5 supports thermogenesis; its loss reduces adipose browning and the expression of thermogenic genes like UCP1, CIDEA, COX7A1, CPT1b, and MCAD [173]. Overall, SIRT5 promotes oxidative and thermogenic functions while limiting lipid storage.

SIRT6 is a central regulator of AT browning and thermogenesis. It promotes beige and brown fat activation in response to cold or β-adrenergic stimulation, while SIRT6 deficiency reduces UCP1 expression and impairs thermogenic signaling through the p38 MAPK/ATF2 and PGC1α pathways [174]. Loss of SIRT6 in POMC neurons disrupts leptin signaling and diminishes browning and lipolysis in HFD-fed mice [175].

After considering all the evidence of sirtuins being involved in BAT thermogenesis and scWAT browning, we can confidently explore further to gain a deeper understanding of them. However, sirtuins and their analogs have not been studied in humans to the same extent until now. Resveratrol, a SIRT1 activator, has been found to reduce inflammation at high doses, whereas low doses show no metabolic benefits [169]. Pterostilbene and Pinostilbene, methylated derivatives of resveratrol, have been shown to effectively enhance thermogenesis and mitochondrial biogenesis via the SIRT1/PGC1α/SIRT3 axis [176]. Synthetic sirtuin activators, such as SRT501, SRT2104, SRT2379, and SRT3025, are currently under study. However, most of the clinical trials with synthetic sirtuins focus on inflammation and T2DM, while the focus on obesity treatment is yet to be established.

##### Peroxisome-Proliferator-Activated Receptors (PPARs)

PPARs are nuclear transcription factors that regulate lipid metabolism, energy balance, and insulin sensitivity [177]. PPAR family members (α, γ, and δ) show tissue-specific expression and unique metabolic functions [177]. PPARγ tends to increase the size of WAT and contribute to lipid storage. PPARγ agonists can remodel white fat tissue by increasing the number of smaller, metabolically active adipocytes that store lipids more efficiently [178]. These changes are supported by alterations in gene expression that favor lipid uptake and storage and promote the death of enlarged fat cells. Research also points to PPARγ’s role in regulating brown fat function, possibly via mitochondrial activation and browning of fat cells [179]. PPARγ appears to sustain the thermogenic capability of BAT, especially under β3-adrenergic stimulation, but its exact targets in this process remain under investigation.

PPARγ activation boosts UCP1 gene expression for NST in brown fat, while PPARα enhances FAO in the liver, aiding in energy production during fasting, weight loss, and insulin sensitivity [180]. Zfp961, a zinc finger protein expressed in BAT, acts as a negative regulator of thermogenesis by suppressing PPARα activity, and this repression can be reversed by PPARα agonism [181]. This study proves that PPARα is essential for thermogenic activation. Tesaglitazar, which activates both PPARα and PPARγ to differing extents, shows promise in enhancing EE and improving fatty liver [180]. Although less studied than PPARα and PPARγ, PPARδ activation promotes FAO and energy dissipation in muscle and AT. In contrast to PPARγ, which primarily drives lipid storage, PPARδ increases EE and reduces adiposity in preclinical models [182]. Rosiglitazone, a selective PPARγ agonist, has been shown to promote browning in scWAT by activating the PRDM16 pathway [183]. Furthermore, Rosiglitazone and PRDM16 synergistically activate the brown fat genes in vivo by stabilizing PRDM16 protein [183]. PPARγ-targeted therapies can combat obesity by enhancing insulin sensitivity, promoting adipocyte development, and regulating lipid metabolism. However, side effects such as weight gain, swelling, and cardiovascular adverse effects limit their use. Future strategies should develop selective PPARγ modulators or combination treatments to retain the benefits of these therapies while reducing their side effects, thereby promoting safer obesity management.

#### 5.2.5. Others

##### Bone Morphogenetic Protein (BMP) Signaling

Bone morphogenetic proteins (BMPs), also referred to as growth and differentiation factors (GDFs), are part of the larger TGF-β superfamily, which encompasses over 20 identified members. Although initially recognized for bone formation, recent studies highlight their role in thermogenesis and EE whereby they affect BAT and beige adipocyte activity. BMPs (BMP8, BMP7, BMP4, BMP9, and BMP11) promote EE and offer therapeutic potential for obesity and metabolic disorders by activating UCP1 and mitochondrial biogenesis. The primary thermogenic functions and underlying mechanisms of key BMPs are summarized in Table 2.

In obese mice, BMP9 administration reduces hepatic steatosis, serum ALT, and cholesterol levels by increasing BAT thermogenic capacity and promoting browning of scWAT [184]. The heightened FGF21 levels resulting from BMP9 administration were found to contribute to these effects by enhancing FA breakdown in WAT and suppressing lipogenic genes in the liver [52]. Further in vitro studies confirmed that BMP9 supports BAT differentiation and counters the pathological mechanisms of diet-induced obesity by promoting expression of UCP1 and CIDEA [191]. Moreover, intraperitoneal delivery of recombinant BMP9 derivatives in obese mice also prevented excessive weight gain, likely through a reduction in the size of white fat cells [191]. Therefore, there is a pressing need for human studies of BMP9 administration to confirm BMP9’s potential in obesity management.

BMP4, another member of the BMP family, plays a pivotal role in directing the fate and differentiation of adipogenic precursor cells. Specifically, BMP4 secreted by WAT is a key feedback regulator influencing the commitment to either white or beige adipocyte lineages [192]. Although BMP4 promotes adipocyte development, it may suppress the brown fat phenotype during the final stages of differentiation through SMAD-mediated signaling [193]. This effect may also stem from inhibited lipolysis via regulation of hormone-sensitive lipase and diminished PPAR activity [193]. In humans, BMP4 levels correlate positively with adipocyte size [193]. These observations suggest that BMP4 can promote thermogenesis and support an anti-obesity therapeutic strategy, but more evidence is needed before it can be applied to humans.

BMP7 has also emerged as a potential candidate for obesity treatment. AT plays a central role in energy balance, with WAT primarily storing lipids and BAT, as well as beige fat, driving EE through UCP1-mediated heat production [190]. It influences brown fat formation through several pathways, including p38 MAPK, PRDM16, PGC1α, UCP1, and mitochondrial development [190]. Overall, BMPs represent promising targets; however, further research is needed to assess their safety, delivery mechanisms, and tissue-specific effects before clinical application.

BMP8 has two genes, encoded as BMP8a and BMP8b, both of which have been found to be important for fat management. BMP8a has been found to have the ability to inhibit adipogenesis and regulate lipid metabolism [194]. BMP8b can modulate energy metabolism by increasing BAT thermogenesis via the AMPK pathway, which has been further validated by BMP8b knockout mice having a reduced metabolic rate and impaired thermogenesis [186]. BMP8b is also capable of inhibiting adipogenesis via the SMAD2/3 and NF-κB pathways [186]. BMP8 is also associated with BAT thermogenic response in neonates [185]. Human studies are now needed to determine whether BMPs exert similar thermogenic effects on adult brown and beige AT.

##### Interleukins

Beyond their classical roles in inflammation and immunity, several interleukins (ILs) activate brown and beige AT, thereby increasing heat production and EE [195]. IL-6 is the most potent cytokine for thermogenesis. In humans, it drives mitochondrial biogenesis, UCP1 expression, lipolysis, and lipid oxidation in brown/beige fat while also stimulating hypothalamic pathways that further enhance EE and weight loss. Loss of IL-6 in the CNS leads to decreased thermogenic gene expression in BAT, reduced EE, and weight gain [195]. Stimulating IL-6/STAT3 pathways may offer therapeutic benefits by encouraging fat browning and reducing adiposity [196]. IL-6, derived from macrophages and adipocytes, activates genes essential for mitochondrial function and thermogenesis [197].

In addition, IL-27 boosts UCP1 expression in fat cells by triggering the p38 MAPK–PGC1α pathway [198]. It has shown therapeutic promise in mouse obesity models and appears to be downregulated in obese individuals, with levels returning to normal after bariatric surgery [198]. IL-17A and γδ T cells (a distinct immune subset that bridges innate and adaptive immunity) stimulate adipose stromal cells to produce IL-33, which sustains regulatory T cells and helps maintain core body temperature during cold exposure [199]. Mice lacking this pathway struggle with temperature regulation [199]. IL-18 or IL-18R KO mice show complex, often conflicting responses to cold and HFD that affect body weight and EE, varying by fat depot and receptor subtype [200]. So, interleukin therapies could potentially address obesity by decreasing fat inflammation, enhancing insulin sensitivity, and increasing heat production. However, applying this approach in clinical settings remains challenging because of cytokine pleiotropy, complex immune–metabolic interactions, and safety concerns. Future approaches should aim for tissue-specific targeting, utilize engineered cytokine variants, and be combined with current metabolic drugs to achieve precise and durable anti-obesity effects.

## 6. Non-Pharmacological Interventions

Non-pharmacological interventions in thermogenesis focus on lifestyle and environmental strategies to enhance the body’s natural heat production and EE. Cold exposure is one of the most studied methods, as it stimulates BAT activity and promotes the browning of WAT through increased NE release. Regular physical exercise also plays a key role in inducing thermogenic hormones like irisin and improving mitochondrial function in muscle and AT. Additionally, dietary modifications such as intermittent fasting or thermogenic foods (e.g., capsaicin, curcumin, berberine) can modestly boost metabolic rate (Table 1). In a very recent study, Lee et al. found that cysteine deprivation triggers AT thermogenesis and weight loss in mice [201]. These approaches offer accessible and sustainable methods to support weight management and metabolic health.

### 6.1. Cold Exposure

Cold-induced thermogenesis (CIT) has been extensively discussed, as it directly elevates the metabolic rate through sympathetic activation of thermogenesis in skeletal muscle and BAT [202]. When animals are exposed to cold for an extended period, sympathetic innervation triggers the recruitment of BAT through hyperplasia, enabling them to adapt to the ambient temperature. Additionally, prolonged cold exposure gives rise to the apparent induction of browning in WAT [203]. Single-cell and single-nucleus transcriptomic profiling reveals that cold-induced thermogenesis involves coordinated remodeling of multiple nonimmune adipose cell types, highlighting extensive intercellular crosstalk [204]. Adipose stem and progenitor cells (ASPCs), mature adipocytes, the endothelium, smooth muscle, and swan cells were found to be involved in WAT browning [204]. Beige adipocytes arise through a defined trajectory from amphiregulin-expressing progenitors via lipid-generating intermediates, alongside expansion of immune-competent adipocyte and endothelial subpopulations [204]. Due to cold exposure, lipolytic enzymes such as adipose triglyceride lipase (ATGL) and hormone-sensitive lipase (HSL) break down long-chain FAs and trigger thermogenesis [205]. Cold exposure has also been found to increase glucose utilization in rat BAT [206]. The improvement in 2-deoxyglucose (2-DG) uptake following cold exposure is similar to that observed with β-adrenergic agonists [207]. BAT glucose (2-DG) uptake during cold exposure is strictly UCP1- and β-adrenergic-dependent: it is completely abolished by β-blockers or UCP1 KO [207]. In addition, the substantial rise in mitochondrial protein levels during cold acclimation also enhances the capacity for oxidative phosphorylation [208]. Additionally, cold acclimation enhances peripheral insulin sensitivity in humans [209]. Some animal studies have suggested that cold stress influences thermoregulation and metabolism not only in the exposed individual but also in subsequent generations [207]. Experiencing a cold during late pregnancy can lead to a higher risk of CIT and increased thermogenic activity of BAT in newborns [207]. However, prolonged cold exposure may elevate blood pressure (BP) in susceptible individuals [76]. In humans, a key unresolved question is whether the limited amount of active BAT is sufficient to drive clinically meaningful thermogenesis. Cold exposure is effective for BAT activation but is impractical for humans given clothing and heating systems. Furthermore, chronic exposure carries risks, including increased BP and the induction of atherosclerosis.

### 6.2. Diet

Diet-induced thermogenesis (DIT) is the modifiable increase in EE following a meal, typically accounting for about 10% of total EE. DIT is influenced by factors such as meal composition, food taste, sleep, lifestyle, physical activity, age, and metabolic disorders. DIT tends to decline with age and is typically lower in obese individuals. Hamada et al. reported that chewing increases postprandial DIT [210]. Different thermogenic food ingredients, such as capsaicin, capsinoids, and so on (Table 1), can also induce thermogenesis and EE. The significance of DIT in combating obesity remains a topic of interest for researchers, as it is a crucial tool to manage EE. Like CIT, DIT activates the β-adrenergic pathway, leading to increased BAT thermogenesis by enhancing UCP1 expression. Cold- CIT is almost entirely driven by sympathetic β-adrenergic activation of BAT, whereas DIT in humans depends only partly on the SNS. A significant component is mediated independently by meal-stimulated gut hormones (e.g., GLP-1, CCK) and bile acids (via TGR5) that directly activate BAT and beige fat [211]. Glick et al. suggested that BAT is activated after a single meal and observed that the respiration rate of BAT increased two hours post-food intake in rats [212]. Furthermore, the authors demonstrated that consuming a meal resulted in an increase in UCP1 activation [211]. In addition, a research group found that metabolic activation of BAT in rats after a liquid meal decreased following surgical severing of sympathetic nerves entering BAT [213]. While BAT’s role in DIT has been shown in small rodents, information on its effects in humans remains scarce. Mutations in β3AR and UCP1 are associated with high fat deposition in the body, a lower metabolic rate, and reduced weight loss following a low-calorie diet [211].

So, dietary and nutrient modulation may play a significant role in fighting obesity and other metabolic disorders through DIT. The circadian rhythm might influence DIT. Timing meals during periods of higher thermogenic responsiveness (morning versus evening) could improve efficiency. However, since the effectiveness of DIT depends on factors like age, sex, body composition, and metabolic health, its performance in obese humans remains uncertain.

### 6.3. Exercise

Exercise is one of the most potent physiological activators of EE and a cornerstone of metabolic health. Both acute and chronic physical activity profoundly influence AT remodeling, mitochondrial function, and whole-body substrate metabolism, making exercise a powerful non-pharmacological tool for obesity management [214]. A single bout of moderate-to-vigorous exercise rapidly increases circulating catecholamines, free FAs, and myokines (e.g., irisin, meteorin-like 1, BAIBA, FGF21), many of which directly stimulate brown and beige adipocyte thermogenesis [215,216]. Irisin, cleaved from FNDC5 in skeletal muscle, crosses into the circulation and induces UCP1 expression and CIDEA expression in WAT via p38 MAPK and ERK signaling, promoting browning in rodents and humans [101,217]. Similarly, meteorin-like 1 drives eosinophil-dependent IL-4 release, which recruits alternatively activated macrophages that further support beige fat development [218].

In humans, the picture is more nuanced. Endurance-trained athletes and regularly active individuals exhibit higher BAT volume and ^18^F-FDG uptake during cold exposure than sedentary controls [219,220]. High-intensity interval training (HIIT) and moderate continuous training both increase cold-induced EE and circulating irisin levels [221]. However, direct evidence of exercise-induced browning in human scWAT remains controversial. Several biopsy studies (10–16 weeks of endurance or resistance training) have failed to detect significant increases in UCP1, CIDEA, or TMEM26 mRNA in abdominal or femoral scWAT [222,223]. In contrast, more recent reports using improved beige markers (e.g., CITED1, CD137) or longer training durations (>6 months) have observed modest browning in gluteofemoral depots, particularly in women and younger individuals [224,225].

Exercise also enhances UCP1-independent thermogenesis in skeletal muscle through futile calcium cycling (SERCA–sarcolipin) and creatine-driven substrate cycling, mechanisms that become especially relevant in obese or aged individuals with reduced BAT mass [226].

In summary, while exercise robustly activates existing BAT and secretes browning factors in humans, classical “beiging” of white fat appears weaker and more variable than in rodents. Nevertheless, the combined effects on BAT recruitment, muscle thermogenesis, and overall EE make regular physical activity an indispensable component of any thermogenesis-based anti-obesity strategy.

### 6.4. Circadian Regulation of Adipose Thermogenesis

AT thermogenesis is under powerful circadian control at multiple levels: central (suprachiasmatic nucleus), peripheral (adipocyte-intrinsic clocks), and systemic (behavioral timing of light, food, and activity). In brown and beige adipocytes, the core molecular clock machinery, comprising the transcriptional activators BMAL1 and CLOCK and the repressors PER1/2 and CRY1/2, drives 24 h oscillations of key thermogenic and lipolytic genes, including UCP1, PGC1α, DIO2, PPARα, PPARγ, ATGL, HSL, and LPL [227,228,229,230]. The nuclear receptors REV-ERBα/β and RORα/γ link the core clock to energy metabolism: REV-ERBα directly represses UCP1, DIO2, and mitochondrial biogenesis genes, whereas RORα activates them [229,231].

Genetic disruption of the adipose clock dramatically alters thermogenic capacity. Adipocyte-specific BMAL1 KO abolishes rhythmic UCP1 expression, severely impairs CIT, and predisposes mice to obesity even when they are fed normal chow [232]. Conversely, deletion or pharmacological antagonism of REV-ERBα (using SR9009 or SR9011) markedly increases UCP1 levels, mitochondrial density, BAT recruitment, and 24 h EE while protecting against diet-induced obesity and glucose intolerance [233,234,235].

In humans, BAT exhibits pronounced diurnal rhythmicity. ^18^F-FDG uptake and tissue perfusion are highest between 08:00 and 12:00 and lowest in the late evening [236,237]. Consequently, mild cold exposure (16–19 °C) in the morning elicits 30–50% greater NST and oxygen consumption than identical exposure in the evening, a difference that disappears in clock-disrupted models [232,236]. Morning cold also preferentially recruits beige adipocytes in subcutaneous depots [238].

Meal timing is another potent synchronizer of the adipose clock. Late calorie intake (after 20:00) suppresses DIT, reduces postprandial BAT activation, and blunts morning UCP1-dependent EE [236,239]. In contrast, early time-restricted feeding (e.g., an eating window of 08:00–16:00) increases beige fat markers, enhances daily lipid oxidation, and improves insulin sensitivity in overweight adults [240,241,242].

Chronic circadian misalignment—common in shift workers, night eaters, and short sleepers (<6 h/night), markedly reduces BAT volume, UCP1 expression, and cold-induced EE while increasing obesity and T2D risk [243,244,245]. Restoration of circadian alignment through consistent sleep schedules, morning bright-light exposure, early meal timing, and morning exercise rapidly reactivates BAT and potentiates the thermogenic effects of cold and physical activity [246].

Thus, circadian optimization represents a completely non-invasive, zero-cost adjuvant strategy. Aligning cold exposure, meals, and exercise with the biological morning (when the thermogenic program is naturally upregulated) can significantly amplify the efficacy of all other non-pharmacological interventions. It should be incorporated into future lifestyle-based obesity management programs.

## 7. Conclusions and Future Perspectives

It is certain that both in mammals and animals, BAT and beige AT play major roles in NST. They help boost EE, improve glucose uptake, promote lipolysis, and support lipid oxidation, all of which are vital in fighting obesity. However, caution must prevail when extrapolating findings from rodent models to humans, as thermogenic regulation involves multiple pathways that differ in their tissue specificity, UCP1 dependence, and functional roles as direct or permissive effectors (Table 3). We have also observed that pharmacological approaches mainly act as direct thermogenic effectors, whereas non-pharmacological interventions primarily serve as permissive and maintenance pathways that progressively enhance thermogenic capacity (Table 3). Rodents keep more BAT in adulthood and are often kept below their thermoneutral zone, encouraging a stronger thermogenic response. In terms of adrenergic signaling, rodents appear to be more dependent on β3-adrenergic activation compared to humans, who rely more on β2-adrenergic signaling. Although UCP1 shows high structural homology across species, subtle sequence differences determine how regulatory transcription factors bind and affect the extent of thermogenesis [247]. Major translational hurdles for adrenergic-targeted thermogenesis include lower BAT mass in humans, reliance on β2-signaling (vs. rodent β3), and cardiovascular side effects that limit dosing.

GLP-1 agonists are well known for weight loss with minimal side effects, showing 15–25% weight reduction over a year [248]. The most well-known of this class of drugs are injectable liraglutide and semaglutide, which act by suppressing appetite, inhibiting glucagon secretion, and delaying gastric emptying [249,250]. To improve compliance, a change in dosage form (oral semaglutide; 2.5 mg) has also shown similar efficacy in terms of weight loss in clinical trials [251]. Obesity’s complexity causes varied drug responses, especially with co-morbidities like T2D. GLP-1 agonists often reach a weight loss plateau despite continued use. To promote sustainable weight loss and preserve lean mass, the focus has shifted to targeting hormones such as GIP and amylin, in addition to GLP-1, using dual and triple agonists. Tirzepatide is the first in its class to be approved as a dual agonist (GLP-1 and GIP), with many others undergoing phase 3 clinical trials. Survotide and Mazdutide are both weekly GLP-1 and glucagon agonists that have shown promising results for weight loss in obese and diabetic participants, respectively [252]. Cagrisema, a newer candidate, is a combination of a GLP-1 agonist (semaglutide) and an amylin analog (Cagrilintide), which in clinical trials has shown an average weight loss of around 20–22% of body weight in participants over a span of 68 weeks [253]. To overcome the plateau effects of centrally acting GLP-1 agonists, it is warranted to investigate co-dosing strategies with drugs that directly target EE and thermogenesis.

Although UCP1-dependent mechanisms—such as activation via the CNS and GLP-1 agonists—remain central to obesity research, they primarily target BAT. It is also crucial not to overlook UCP1-independent mechanisms, which represent major non-adrenergic pathways capable of direct heat generation and are primarily found in brite/beige adipocytes and skeletal muscle, together constituting a much larger mass in the human body (Table 3). The contribution of UCP1-independent thermogenesis remains poorly quantified in humans. Among these pathways, the SERCA2b activator CDN1163 has shown preclinical promise by reducing fat mass and improving glucose tolerance in obese and diabetic mice [254]. Recently, an endoplasmic reticulum (ER)-membrane-anchored peptide, C4orf3, has been discovered as a molecular resistor of SERCA2b [255]. Absence of this peptide reduced thermogenesis by SERCA2b and increased body mass in rats. A natural compound, Celastrol, was found to bind to creatine kinase B-type in human BAT, thereby stabilizing it and dissipating heat via creatine futile cycling [256]. However, this drug faces clinical limitations due to its poor bioavailability and low ceiling for toxicity. A small-molecule inhibitor, CX-4945, also reduced weight gain in obese mice by preventing the maturation of fat cells by stabilizing C/EBPβ, indirectly preventing the activation of PPARγ [257]. Activating creatine or calcium cycling can lead to unintended EE (potential cachexia), mitochondrial stress, muscle wasting, and calcium ion-related irregularities. Another UCP1-independent approach undergoing a phase I clinical trial involves the targeted ileo-colonic delivery of conjugated bile acids with an affinity to TGR5, ultimately leading to stimulation of GLP-1 secretion and weight loss [258]. GDF15 is crucial for both UCP1-dependent and -independent thermogenesis, and it increases satiety and impacts food intake via CNS activity. LY3463251, a fusion protein agonist of GDF15 undergoing a phase I trial, showed a reduction in body weight of up to 3%, with many more drugs targeting this stress-induced cytokine [259]. Thus, multimodal therapy combining various forms of non-adrenergic interventions, especially for a multifactorial condition such as obesity, seems promising.

Emerging evidence further highlights the pivotal role of circadian rhythmicity in human adipose thermogenesis. Brown and beige fat activity, cold-induced EE, and DIT all exhibit strong diurnal variation, peaking in the morning and declining toward the evening. Chronic circadian disruption markedly suppresses BAT function and increases obesity risk, whereas aligning meals, exercise, and cold exposure with the early active phase significantly enhances thermogenic capacity. Incorporating chronobiology-based timing into both pharmacological and lifestyle interventions, therefore, represents a simple, cost-free strategy to maximize EE and improve long-term weight-loss outcomes.

While developing and refining various drug delivery platforms for complex conditions like obesity is crucial, it is equally important to explore beyond well-known targets through unbiased screening. Adipocytes are dynamic organelles that communicate with other metabolic organs via secreted cargoes (hormones, extracellular vesicles, microRNAs), guiding downstream signaling and profoundly affecting thermogenesis and weight loss. Using CRISPR/Cas9 to characterize smORFs (small mitochondrial open reading frames) and their encoding microproteins allows screening for lipid metabolism regulators and directly pinpoints druggable treatment targets [260]. With the advent of AI tools, for example, AlphaFold, screening structural proteins and predicting drug/protein and protein/protein interactions have become much faster processes, aiding in the drug development pipeline. Another aspect of treating obesity lies in diet; many of the above-mentioned therapeutic options affect gut health and microbiome. Responses to obesity therapies vary among individuals, prompting the exploration of personalized approaches that consider the gut microbiome. Ongoing research is investigating how the microbiome influences therapy response and how anti-obesity treatments affect the microbiome through pathways linked to thermogenesis. However, microbiome-based personalization remains experimental and requires validation using specific stratification variables and clinically relevant endpoints. Thus, effective obesity management requires a multipronged, patient-centered strategy that integrates evidence-based interventions with individual profiles, clinical context, and long-term adherence.

## Figures and Tables

**Table 1:** Thermogenic mechanisms of natural and synthetic compounds.

Compound	Type	Study Model	Mechanism in Thermogenesis	Primary Targets/Pathways	References
Mirabegron	Small molecule	Human	Activates BAT via β3-adrenergic stimulation	β3-adrenergic receptors ⟶ ↑ cAMP ⟶ PKA ⟶ UCP1	[6,9]
CL316,243	Small molecule	Mouse	Selective β3-adrenergic agonist	β3-adrenergic ⟶ ↑ cAMP ⟶ PKA ⟶ UCP1	[43]
Formoterol	Small molecule	Human	Non-selective β-adrenergic agonist	β2/β3-adrenergic activation	[47]
GLP-1 analogs (liraglutide, semaglutide)	Peptide	Mouse, Human	Promotes browning of WAT and activates BAT	GLP-1R activation ⟶ ↑ SNS outflow	[48,49,50]
FGF21	Peptide hormone	Mouse	Enhances BAT activity and WAT browning	FGFR1c/β-Klotho ⟶ PGC1α, UCP1	[51,52]
Irisin	Myokine (peptide)	Mouse, Human	Converts WAT to beige fat	AMPK–PGC1α pathway ⟶ ↑ UCP1	[53]
Capsinoids	Small molecule	Human	Activates BAT via TRPV1	TRPV1 ⟶ ↑ SNS ⟶ β-adrenergic signaling	[54]
Salsalate	Small molecule	Mouse	Mild mitochondrial uncoupler	Inhibits NF-κB, ↑ mitochondrial respiration	[40]
Capsaicin	Natural compound	Mouse, Human	Activates SNS to increase EE	TRPV1 activation ⟶ ↑ catecholamines ⟶ β-adrenergic signaling	[55]
Caffeine	Natural compound	Human	Stimulates CNS ⟶ ↑ catecholamines ⟶ thermogenesis	Adenosine receptor antagonist, ↑ cAMP	[56]
Resveratrol	Natural compound	Mouse	Activates mitochondrial biogenesis and BAT genes	SIRT1-PGC1α, AMPK	[57]
Curcumin	Natural compound	Mouse	Promotes browning of WAT	AMPK, PPARγ coactivator pathways	[58,59]
Gingerol	Natural compound	Mouse	Activates sympathetic thermogenesis	TRPV1 activation, AMPK	[60]
Fucoxanthin	Natural compound	Mouse	Promotes UCP1 expression in WAT and BAT	β3-adrenergic and PGC1α pathways	[61]
Naringin	Natural compound	Human	Increases metabolic rate via β-adrenergic stimulation	AMPK, PPARγ coactivator pathways	[62]
Menthol	Natural compound	Mouse, Human	Activates cold-sensing TRPM8 ⟶ BAT activation	TRPM8–SNS–β-adrenergic pathway	[63,64]
Quercetin	Natural compound	Mouse	Enhances BAT activity and browning of WAT	AMPK, PGC1α, bile acid	[65,66]
Berberine	Natural compound	Mouse	Promotes BAT activation and WAT browning	AMPK–SIRT1–PGC1α pathway	[67]
Baicalein	Natural compound	Mouse	Induces beige adipogenesis and BAT thermogenesis	AMPK–SIRT1–PGC1α pathway	[68]
Retinoic acid/β-carotene	Small molecule	Mouse	Stimulates UCP1 expression in BAT	RAR/RXR signaling ⟶ ↑ PGC1α and Ca^2+^ cycling	[69]
TUG-891	Small molecule	Mouse	Activates BAT and browning of WAT	GPR120 (FFA4) agonist ⟶ ↑ p38 MAPK, AMPK ⟶ UCP1	[70]

**Table 2:** Primary thermogenic functions and underlying mechanisms of different BMPs in AT.

BMP	Primary Function in Thermogenesis	Mechanism/Pathway	Study Model	References
BMP9	Enhances BAT thermogenesis and metabolism	SMAD-dependent; ↑ oxidative metabolism	Mouse	[184]
BMP8	Enhances BAT thermogenesis and sympathetic innervation	Central (hypothalamic) and peripheral action; ↑ NE sensitivity	Mouse, Human	[185,186]
BMP4	Induces beige adipocyte formation in WAT	SMAD-dependent; upregulates thermogenic genes	Mouse	[187]
BMP2	Modulates adipocyte commitment toward beige phenotype	BMP2–SMAD signaling; synergistic with BMP4	Mouse	[188]
BMP7	Promotes brown adipocyte differentiation and thermogenesis	Activates p38 MAPK and SMAD1/5/8 ⟶ ↑ UCP1	Mouse, Human	[189,190]

**Table 3:** Summary of primary sites of action and mechanistic roles of thermogenic receptors and pathways.

Receptor/Pathway	Primary Location(s)/Action	Core Mechanism	Evidence Base	UCP1 Dependence	Thermogenic Role	Refs.
β3-AR	BAT, beige AT	cAMP–PKA–p38-UCP1	Rodent, Human	UCP1-dependent	Direct	[6,9]
β2-AR	Lungs, blood vessels, uterus, AT	cAMP–PKA–p38-UCP1	Human	UCP1-dependent	Direct	[47]
β1-AR	Heart, kidneys, AT	cAMP–PKA–p38-UCP1	Rodent, Human	UCP1-dependent	Supportive	[79]
α1-AR	Vascular smooth muscle, brain, AT	Ca^2+^ influx–cAMP potentiation	Rodent	Both	Supportive	[69,71]
α2-AR	CNS, AT	↓ SNS outflow	Rodent	UCP1-dependent	Inhibitory	[82]
GLP1R	Pancreas, lungs, gut, brain, liver, AT	CNS AMPK inhibition-SNS	Rodent, Human	UCP1-dependent	Permissive	[48,49,50]
NPRs (NPRA/ NPRC)	Heart, brain, lungs, gut, AT	cGMP–PKG–p38-UCP1	Rodent, Human	UCP1-dependent	Direct and Supportive	[92]
FGF21	Liver, AT	PGC-1α, lipid oxidation	Rodent, Human	UCP1-independent	Direct	[96]
Irisin	Skeletal muscle, AT	AMPK–PGC-1α-browning and UCP1 induction	Rodent, Human	UCP1-dependent	Permissive	[101,103]
Leptin	AT	SNS activation, thermogenic activation	Rodent, Human	Indirect	Permissive	[106,107,108]
GPR3	Brain, BAT	Constitutive Gs-cAMP	Rodent, Human	UCP1-dependent	Direct	[113]
TGR5	Intestine, AT	cAMP–PKA–DIO2, FCC	Rodent, Human	Both	Direct and Permissive	[116]
FXR	Gut, liver, AT	Nuclear receptor modulation	Rodent	UCP1-dependent	Inhibitory	[125]
TRPV1	Gut, skin, AT	Ca^2+^–AMPK–SIRT1-UCP1	Rodent, Human	UCP1-dependent	Permissive	[55]
TRPV2	Lymphocytes, macrophages, neurons, AT	Ca^2+^ influx synergizes β-AR	Rodent	UCP1-dependent	Supportive	[130]
TRPV4	AT, smooth muscle, endothelium	Inhibits p38MAPK	Rodent	UCP1-dependent	Inhibitory	[131]
TRPM8	BAT, sensory nerves	Cold-sensing SNS-β-AR	Rodent, Human	UCP1-dependent	Permissive	[63,64]
SERCA–Sarcolipin Ca^2+^ Cycling	Skeletal muscle, beige AT	ATP hydrolysis futile cycling	Rodent	UCP1-independent	Direct	[140]
Creatine Futile Cycle	BAT, beige AT	ATP-consuming creatine cycling	Rodent	UCP1-independent	Direct	[142]
TRα/TRβ	Brain, heart, AT, skeletal muscle	T4 ⟶ T3 (DIO2)-β3/SRT1	Rodent, Human	UCP1-dependent	Permissive and Direct	[145]
Estrogen Receptors (Erα/β)	Brain, AT	SNS + TGF-β modulation	Rodent, Human	Both	Permissive	[155]
Growth Hormone Receptor (GHR)	Liver, AT, brain	MEK-ERK pathway	Rodent, Human	UCP1-dependent	Permissive	[162]
Sirtuins (SIRT1, SIRT3)	BAT, WAT, muscle, mitochondria	Deacetylation of PGC-1α, mitochondrial biogenesis, FA oxidation	Rodent, Human	UCP1-dependent	Permissive	[169]
PPARs (PPARα, PPARγ, PPARδ)	WAT, BAT, liver, skeletal muscle	Mitochondrial biogenesis, FA oxidation	Rodent, Human	UCP1-dependent	Permissive	[180]
BMPs	AT, kidneys, brain, liver, prostate	SMAD + p38 ⟶ mitochondrial genes	Rodent, Human	UCP1-dependent	Permissive and Direct	[190]
Interleukins	Immune cells, AT	SMAD + p38 ⟶ mitochondrial genes	Rodent	UCP1-dependent	Supportive	[198]
Cold Exposure	Brain, BAT, beige AT, skeletal muscle	SNS activation-β-AR-UCP1	Rodent, Human	UCP1-dependent	Direct	[203]
Exercise Training	Skeletal muscle, AT	Myokines (irisin), SNS activation, mitochondrial remodeling	Rodent, Human	Both	Permissive	[215,216]
Diet-Induced Thermogenesis	Gut, brain, AT	SNS activation, gut hormones, nutrient sensing	Rodent, Human	UCP1-dependent	Supportive	[211]
Circadian Regulation	Gut, brain, AT	Metabolic genes, SNS tone, mitochondrial function	Rodent, Human	Both	Permissive	[227,228,229,230]

## Data Availability

No new data were created or analyzed in this study.

## Abbreviations

The following abbreviations, in alphabetical order, are used in this manuscript:

ADP	Adenosine Diphosphate
ALT	Alanine Aminotransferase
AMPK	AMP-Activated Protein Kinase
ANP	Atrial Natriuretic Peptide
AT	Adipose Tissue
ATP	Adenosine Triphosphate
BAT	Brown Adipose Tissue
BAIBA	Β-aminobutyric Acid
BMP	Bone Morphogenetic Protein
BMPR	Bone Morphogenetic Protein Receptor
BMR	Basal Metabolic Rate
BNP	B-Type Natriuretic Peptide
cAMP	Cyclic Adenosine Monophosphate
CD	Cluster of Differentiation
cGMP	Cyclic Guanosine Monophosphate
CIDEA	Cell-Death-Inducing DNA Fragmentation Factor α-Like Effector A
CIT	Cold Induced Thermogenesis
CITED1	Cbl-interacting Protein Expression to Differentiation 1
CNP	C-Type Natriuretic Peptide
CNS	Central Nervous System
CREB	Camp Response Element-Binding Protein
CVD	Cardiovascular Disease
DACRA	Dual Amylin and Calcitonin Receptor Agonists
2-DG	2-Deoxyglucose
DIO2	Type 2 Deiodinase
DIT	Diet-Induced Thermogenesis
DMH	Dorsomedial Hypothalamus
EPAC1	Exchange Protein Activated by cAMP 1
ERα/β	Estrogen Receptor α/Β
FA	Fatty Acid
FAO	Fatty Acid Oxidation
FCC	Futile Creatine Cycle
FFA	Free Fatty Acid
FGF21	Fibroblast Growth Factor
FXR	Farnesoid X Receptor
GC	Guanine–Cytosine
GDF15	Growth Differentiation Factor 15
GH	Growth Hormone
GIP	Glucose-Dependent Insulinotropic Polypeptide
GIT	Gastrointestinal Tract
GLP-1	Glucagon Like Peptide-1
GPR3	G-Protein-Coupled Receptor 3
GTP	Guanosine Triphosphate
HFD	High-Fat Diet
HSL	Hormone-Sensitive Lipase
IL	Interleukins
IR	Insulin Resistance
KCNK3	Potassium Channel, Subfamily K, Member 3
KO	Knockout
MAPK	Mitogen-Activated Protein Kinase
MetAP2	Methionine Aminopeptidase 2
mTOR	Mammalian Target of Rapamycin
NAD	Nicotinamide Adenine Dinucleotide
NE	Norepinephrine
NPR	Natriuretic Peptide Receptor
NST	Non-Shivering Thermogenesis
OMM	Outer Mitochondrial Membrane
OXPHOS	Oxidative Phosphorylation
PAT2	Proton-coupled Amino Acid Transporter 2
PGC1α	Peroxisome-Proliferator-Activated Receptor Gamma Coactivator 1-Alpha
PKA	Phosphokinase A
PKG	Phosphokinase G
PPAR	Peroxisome-Proliferator-Activated Receptor
PRDM	Positive Regulatory Domain
ROR α/γ	Retinoic Acid-related Orphan Receptors α/γ
Sca1	Stem Cell Antigen 1
scWAT	Subcutaneous White Adipose Tissue
SERCA	Sarco/Endoplasmic Reticulum Calcium ATPase Protein
SIRT	Sirtuin
SLN	Sarcolipin
SMAD	Small Mother Against Decapentaplegic
SNA	Sympathetic Nerve Activity
SNS	Sympathetic Nervous System
SVF	Stromal Vascular Fraction
T2DM	Type 2 Diabetes Mellitus
T3	Triiodothyronine
T4	Thyroxine
TBX1	T-Box Transcription Factor 1
TG	Triglycerides
TGF-β	Tumor Growth Factor-β
TGR5	Takeda G-Protein-Coupled Receptor 5
TH	Thyroid Hormone
TMEM	Transmembrane Protein
TNF-α	Tumor Necrosis Factor-α
TRα/β	Thyroid Receptor α/Β
TRP	Transient Receptor Potential
TRPM	Transient Receptor Potential Melastatin
TRPV	Transient Receptor Potential Vanilloid
UCP1	Uncoupled Protein 1
VMH	Ventromedial Nucleus of the Hypothalamus
vWAT	Visceral White Adipose Tissue
WAT	White Adipose Tissue
Zfp	Zinc Finger Protein
α/βAR	α/βAdrenergic Receptor
